# FeMoS_2_ micoroparticles as an excellent catalyst for the activation of peroxymonosulfate toward organic contaminant degradation†

**DOI:** 10.1039/d3ra00707c

**Published:** 2023-03-17

**Authors:** Cai-Wu Luo, Lei Cai, Chao Xie, Jing Wu, Tian-Jiao Jiang

**Affiliations:** a Research Center for Eco-Environmental Sciences, Chinese Academy of Sciences 100085 China luocaiwu00@126.com +86-734-8282345; b School of Resource Environmental and Safety Engineering, University of South China 421000 China; c Ningxia Modern Construction Technology Vocational Skills Public Training Center, Ningxia College of Construction 750021 China

## Abstract

The FeMoS_2_ catalyst for activating peroxymonosulfate (PMS) is a promising pathway for removing organic pollutants in wastewater, however, the dominant FeS_2_ phases and sulfur (S) vacancies in it are little involved. Herein, for the first time, novel bimetallic FeMoS_2_ microparticles were synthesized by a simple method and then applied for PMS activation for degrading organic pollutants. The catalysts were characterized by several techniques, including X-ray diffraction and X-ray photoelectron spectroscopies. The results revealed that new FeMoS_2_ microparticles containing S vacancies in the main FeS_2_ phases were obtained. FeS_2_ and S vacancies were found to play important roles for activating PMS by radical and nonradical pathways. More Fe^2+^ and Mo^4+^ were formed in the presence of S vacancies, which offered a new strategy for exploring novel heterogeneous catalysts in the activation of PMS for environmental remediation.

## Abstract 2

The degradation process coexisted with homogeneous and heterogeneous catalytic reactions and the latter was dominant.

## Graphical abstract

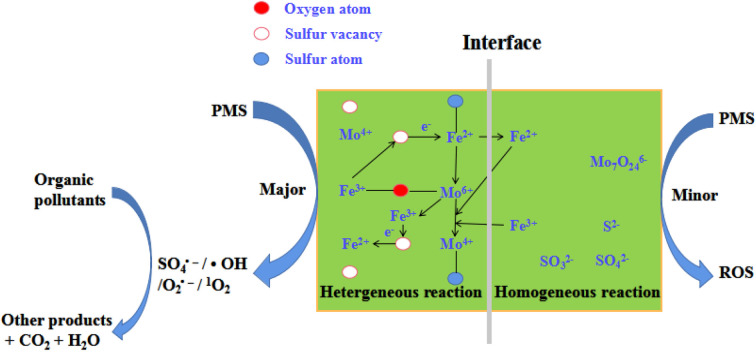

## 1. Introduction

Organic pollutants such as dyes are widely applied in various fields, *e*.*g*., antibiotics,^1^ food additives,^2^ and microplastics,^3^*etc*. However, they are harmful to human health. Consequently, it is extremely necessary to remove them using various advanced oxidation processes.^1–10^ Among these, the Fenton-like technology involving PMS activated by Fe-based catalysts is a good alternative. Over the past years, various Fe-based catalysts have been extensively developed,^5–10^ including single^6,7^ and bimetallic Fe-based catalysts.^5,8–10^ Bimetallic Fe-based catalysts more easily facilitate PMS decomposition by accelerating the transformation of iron(ii) and iron(iii) (Fe^2+^ and Fe^3+^, respectively) due to the function of another metal with variable valence states relative to single Fe-based states (formula (1)). Clearly, the former has more advantages than the latter in PMS activation.

1 Fe^3+^ + M^*n*+^ → Fe^2+^ + M^*n*+1^

To date, research has predominantly focused on Fe–Mn,^8^ Fe–Cu,^5,9^ Fe–Co,^10^ Fe–Ni,^11^ Fe–Ag,^12^ and Fe–Mo.^13–18^ Among these, they are either highly toxicity, such as cobalt and nickel, or expensive, such as silver. It is possible that molybdenum (Mo) better boosts the transformation cycle of Fe^2+^ and Fe^3+^ relative to copper (Cu) and manganese (Mn). This is because the Mo (4d^5^5s^1^) atom accommodates more electrons in empty orbits than Cu (3d^10^4s^1^) and Mn (3d^5^4s^2^) atoms. In addition, Mo has other merits like lower toxicity. Clearly, it is an ideal alternative for Mo as a cocatalyst to promote PMS activation in Fe-based catalysts. Enhancement of the Fe/PMS system has been reported in the assistance of Mo species, such as Mo^0^ (ref. 13) and MoS_2_.^14–18^ Compared to Mo^0^, the exposed Mo^4+^ species in MoS_2_ are more suitable for activating PMS in the presence of unsaturated S species,^14–19^ thereby inducing greater removal of organic pollutants. Recently, some FeMoS_2_ heterogeneous catalysts in PMS-based reactions have been extensively explored, including Fe_3_O_4_@MoS_2_,^20^ FeOOH@MoS_2_,^21^ and Fe–MoS_2_,^22^ which has to overcome the shortcomings of mixtures of FeMoS_2_-based homogeneous and heterogeneous catalysts.^14–17^ However, these processes involve complex procedures. More importantly, most Fe species are deposited on MoS_2_ surfaces, which results in long distances between Fe and Mo atoms, thereby affecting the Fe^2+^/Fe^3+^ cycle. To alter this situation, FeMoS_2_ has been directly prepared by high-temperature treatment of mixtures of Fe, Mo, S, and I powders.^23^ The results show that Fe atoms are confined near Mo atoms and thus interatomic distances turn shorter, which is better for the adsorption and decomposition of PMS. However, the Fe content is extremely low inside the catalyst in this case, restricting larger applications. Nevertheless, this situation has provided a good inspiration for the synthesis of a novel FeMoS_2_ with shorter distances between Fe and Mo atoms using *in situ* synthesis. Interestingly, researchers have found that small crystal phases of FeS_2_ appear in FeMoS_2_*via* the above method.^24,25^ It has been confirmed that FeS_2_ can effectively activate PMS to remove organic pollutants in wastewater,^26,27^ even having a higher ability relative to MoS_2_ alone in Fenton-like reaction.^28^ It has been expected to be able to synthesize FeS_2_ as dominant phases in FeMoS_2_-based materials to enhance catalytic activity. Also, this catalyst would be rich in S atoms. In this case, S vacancies are easily generated in these materials by suitable treatment.^19,29^ Wu *et al.*^29^ have demonstrated that the S vacancies play key role in activating PMS in the Fe(iii)/CoS_2_ system. Thus, it is promising to further strengthen the catalytic performance in FeMoS_2_. There is no doubt that the development of a new FeMoS_2_ containing S vacancies with main FeS_2_ phases is highly desired. To the best of our knowledge, the synthesis of this catalyst and its application for activating PMS to degrade organic pollutants has not been reported so far.

In this study, a new FeMoS_2_ catalyst, containing S vacancies and FeS_2_ as the dominant phases, was synthesized by a one-step method, which could efficiently activate PMS to degrade organic contaminants. Important influencing factors were studied in detail. A different reaction mechanism was found in this FeMoS_2_ for PMS activation. The relationship between the catalyst structure and catalytic performance was established.

## 2. Experiments

### 2.1. Chemical reagents and materials

The chemical reagents and materials were supplied by commercial purchase. For instances, peroxymonosulfate (2KHSO_5_·KHSO_4_·K_2_SO_4_), 5,5-dimethyl-1-pyrro-line*N*-oxide (DMPO, 98%), and 2,2,6,6-tetra-methylpiperidine (TEMP, 99%) were purchased from Sigma-Aldrich, Inc. (St Louis, MO, USA). Molybdenum(iv) sulfide (MoS_2_, ≥98.0%), furfuryl alcohol (C_5_H_6_O_2_, ≥98.0%), ammonium molybdate tetrahydrate ((NH_4_)_6_Mo_7_O_24_·H_2_O, ≥99.0%), and rhodamine B (RhB) were purchased from Sinopharm Chemical Reagent Co., Ltd. (Shanghai, China). Tiron (C_6_H_7_NaO_8_S_2_, >98.0%), sodium sulfide (Na_2_S·9H_2_O, >98.0%), and methyl orange (MO) were obtained from Damao Chemical Reagent Factory (Tianjin, China). Molybdenum trioxide (MoO_3_, ≥99.5%) and methylene blue (MB) were purchased from Kemiou Chemical Reagent Co., Ltd. (Tianjin, China). All the above-mentioned chemical reagents and materials were of analytical grade and employed without further purification.

### 2.2. Synthesis of FeMoS_2_ catalysts

(i) *In situ* synthesis: 0.18 g or 0.94 g or 1.98 g of Fe(NO_3_)_3_·9H_2_O, 2.28 g of CH_4_N_2_S, and 0.196 g of (NH_4_)_6_Mo_7_O_24_·4H_2_O were mixed in 35 mL of distilled water and then evenly stirred. Afterward, the solutions were then autoclaved at 180 °C for 4 h. Then, they were dried at 60 °C and denoted as FeMoS_2_-IS-60. Finally, dried samples were calcined at different temperatures (200, 300, 400, or 500 °C) for 4 h with a heating rate of 5 °C min^−1^ under air atmosphere, thus obtaining the targeted products, respectively denoted as FeMoS_2_-IS-200, FeMoS_2_-IS-300, FeMoS_2_-IS-400, and FeMoS_2_-IS-500. Herein, the theoretic concentrations of Fe in the above catalysts were 1.0, 5.0 and 10 wt%, respectively. Unless otherwise indicated, FeMoS_2_-IS-400 was abbreviated as FeMoS_2_-IS and the concentration of Fe was fixed at 5.0 wt%. The MoS_2_-IS, Fe–Mo, and Fe–S catalysts were synthesized by the same above procedures for FeMoS_2_-IS except for one of components. MoS_2_-300 was obtained by heating commercial MoS_2_ powders at 300 °C for 4 h. The synthetic steps for FeMoS_2_-IS microparticles are displayed in Fig. S1.†

(ii) Post treatment: 0.19 g of Fe(NO_3_)_3_·9H_2_O and 0.50 g of MoS_2_ were weighed and added separately to 50 mL of distilled water. After being evenly stirred, they were autoclaved at 180 °C for 4 h, then cooled to room temperature, and dried at 60 °C. Finally, the dried sample was treated at 400 °C for 4 h with a heating rate of 5 °C min^−1^ under air atmosphere. The targeted product was denoted as FeMoS_2_-IE (theoretic Fe concentration, 5.0 wt%).

### 2.3. Characterization of catalysts

The crystal phases of catalyst were analyzed by using (XRD) spectroscopy with a D8-Advance X-ray Diffractometer (Bruker Corp., Billerica, MA, USA). The specific surface area and pore features of catalyst were measured by liquid nitrogen physisorption using an Autosorb-iQ/ASAP 2460 apparatus (Quantachrome Instruments Corp., Boynton Beach, FL, USA). The morphologies of catalysts were examined by scanning electron microscopy (SEM) on an SU-8100 instrument (Hitachi Instruments, Inc., Tokyo, Japan). Fourier transform-infrared spectroscopy (FT-IR) was measured on a Spectrum 100 (PerkinElmer, Inc., Waltham, MA, USA). Raman Spectroscopy was recorded using an LabRAM HR800 (Horiba Ltd., Tokyo, Japan). The surface elemental information of catalysts was characterized by X-ray photoelectron spectroscopy (XPS) on an EscaLab 250xi instrument (Thermo Fisher Scientific Inc., Pittsburgh, PA, USA). The thermogravimetry (TG) was carried out on the TA SDT650/STA 449 F3 instrument. The vacancies of catalyst and reactive oxygen (O) species were measured by electron paramagnetic resonance (EPR) on a Bruker A300 (Bruker Corp). The zeta potential value was measured on Zetasizer Nano ZS90 (Malvern Instruments Ltd., Malvern, UK).

### 2.4. Removal of contaminants in different reaction processes

Degradation reactions were conducted under atmospheric pressure and room temperature. First, a 10 mg L^−1^ RhB solution was prepared using distilled water. Then, the catalyst together with PMS was immediately added into the solution to maintain the suitable concentration of each component. After addition, the removal reaction proceeded and, at intervals, samples collected, with methanol added to the samples to capture reactive oxygen species (ROS). The absorbance of RhB was measured by spectrophotometer and pH recorded using a pH meter before and after reaction. According to experimental requirements, the important reaction parameters and the related chemical reagents were timely adjusted under optimal conditions. The decomposition of PMS was quantified by the potassium iodide method.^30^ The concentration of Fe^2+^ was quantified using the 1,10-phenanthroline method, with Fe^3+^ first reduced by hydroxylamine hydrochloride and then total Fe^2+^ quantified by the above method.^13–15^

## 3. Results and discussion

### 3.1. Characterization of catalysts

**Fig. 1:**
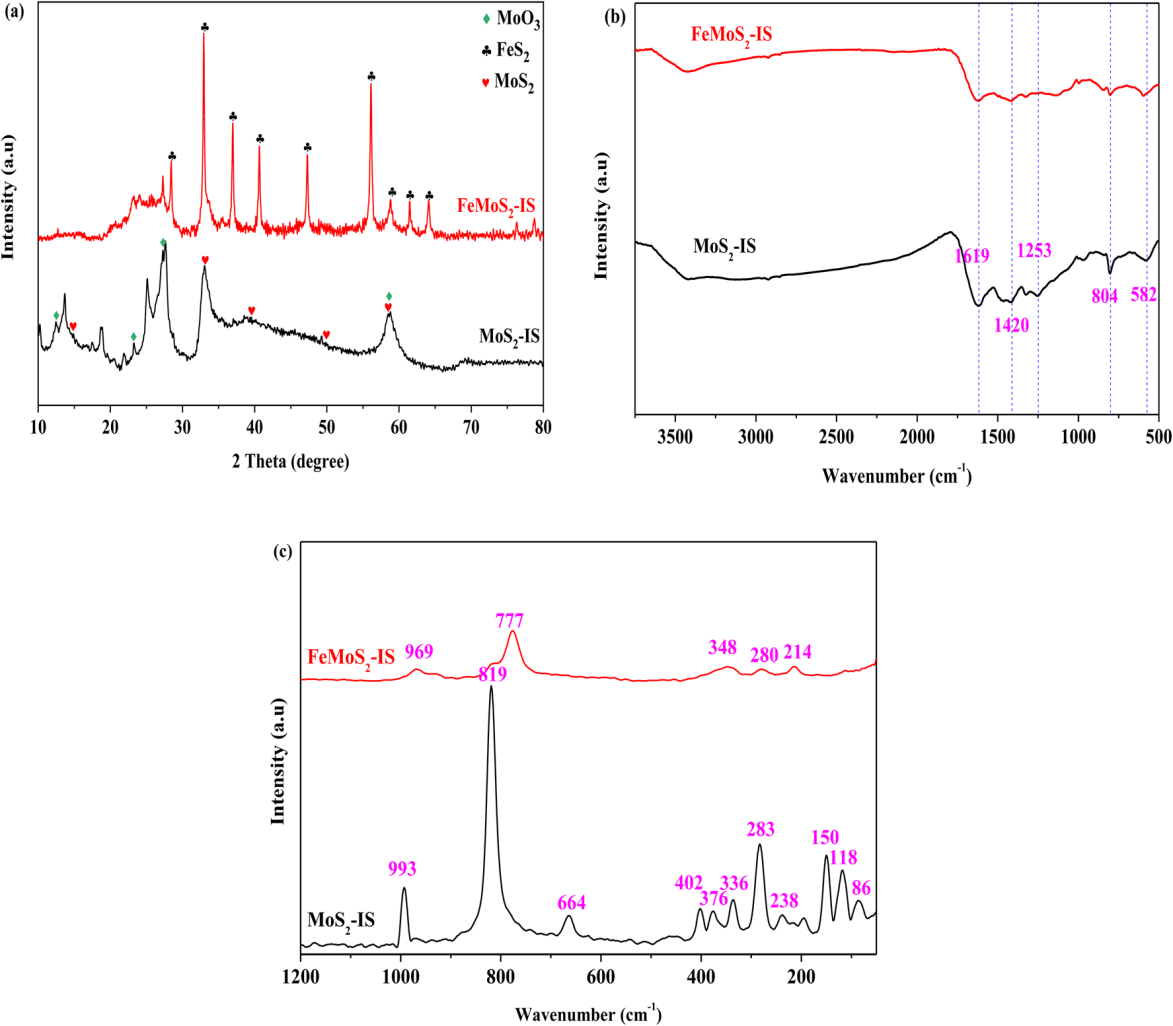
(a) XRD patterns of MoS_2_-IS and FeMoS_2_-IS; (b) FT-IR spectra of MoS_2_-IS and FeMoS_2_-IS; (c) Raman spectra of MoS_2_-IS and FeMoS_2_-IS.

XRD patterns of MoS_2_-IS and FeMoS_2_-IS showed that, for MoS_2_-IS, diffraction characteristic peaks located at 2*θ* = 14.38°, 32.68°, 39.54°, 49.79° and 58.33°, which were attributed to the crystal phases of MoS_2_ (PDF-# 37-1492, Fig. 1a). This originated from the reaction between ammonium molybdate and thiourea during hydrothermal treatment.^21^ Some diffraction characteristic peaks located at 2*θ* = 12.76°, 23.33°, 27.33° and 58.80° were ascribed to the crystal phases of MoO_3_ (PDF-# 05-0508). The introduction of O was derived from the oxidation of MoS_2_ in high-temperature treatment. In addition, introducing O also occurred during hydrothermal treatment.^31^ These results were further confirmed from the Raman spectra (see Fig. 1c). In general, the widths of diffraction characteristic peaks were large in MoS_2_-IS, implying that its crystallization was not high, in other words, it is very difficult to identify the crystal phases of MoS_2_ and MoO_3_. It was agreement with the literature.^20,21^ After modification, the diffraction characteristic peaks of FeMoS_2_-IS were greatly altered relative to those of MoS_2_-IS, namely, some diffraction characteristic peaks disappeared and others newly appeared. On the one hand, this did not present diffraction characteristic peaks of MoS_2_. This was most probably due to it being either highly dispersed on the support or having low crystallization. Simultaneously, MoO_3_ intensity signals were decreased. On the other hand, some clear diffraction characteristic peaks were newly observed. For example, sharp diffraction peaks located at 2*θ* = 36.96°, 40.64°, 47.30°, 56.12°, 58.82°, 61.48° and 64.12° were observed, which were attributed to the crystal phase of FeS_2_ (PDF-# 42-1340).^24^ In other words, the main crystal phases of the catalyst were FeS_2_. As mentioned above, the crystal phases of FeS_2_ were little identified in FeMoS_2_. In fact, FeS_2_ has been employed to degrade organic pollutants in advanced oxidation processes,^26,27,32,33^ but it was not FeS_2_ as dominant phases in the various FeMoS_2_ catalysts for activating PMS.^20–22^ Consequently, FeS_2_ appearance was in favor of the activation of PMS in this reaction.

One wonders whether the synthesis and application of FeS_2_ occurs without the addition of Mo? The answer is No. As evidenced by XRD characterization, the typical diffraction characteristic peaks of samples was mainly attributed to the structure of FeS_2_ (Fig. S2a†). Therefore, the Mo existence was no a prerequisite for FeS_2_ generation but it mainly affected the catalytic performance (see below Fig. 3). In addition, other active sites might have been highly dispersed on the support, such that they were not detected. For instance, with MoS_2_-300 as an indirect evidence, the reaction between S of MoS_2_ and O in air occurred. Thus, S vacancies were formed due to the different atomic radius between S and O, as evidenced from EPR characterization (Fig. S2b†). The result indicated that the relative intensity of MoS_2_-300 was altered relative to those of MoS_2_, hinting that the S vacancies may generate in the FeMoS_2_.^19^

To further explore the differences, SEM was employed and MoS_2_-IS observed to exhibit a plate-like structure with the partially agglomerated appearance (Fig. S3a and b†). After modification, the morphology of FeMoS_2_-IS was greatly different and the catalyst shape became large aggregation particles. The pore structure of MoS_2_-IS and FeMoS_2_-IS showed that, for MoS_2_-IS, the *S*_BET_, *V*_Total_, and *D*_A_ of this catalyst were 12.69 m^2^ g^−1^, 0.0346 cm^3^ g^−1^, and 10.91 nm, respectively (Table 1). After modification to form FeMoS_2_-IS, the *S*_BET_ was clearly reduced, which might be attributed to changed morphology, as evidenced from SEM results. In contrast, the *V*_Total_ and *D*_A_ of FeMoS_2_-IS were increased, which was beneficial for enhancing mass transfer. Combined with the removal of RhB and *S*_BET_, the *S*_BET_ was confirmed not to be an important factor for improving RhB removal.

**Table 1:** Pore structure of different catalyst^a^

Catalysts	*S*_BET_ (m^2^ g^−1^)	*V*_Total_ (cm^3^ g^−1^)	*D*_A_ (nm)
MoS_2_-IS	12.69	0.0346	10.91
FeMoS_2_-IS	7.85	0.0375	19.12

a *S*_BET_, *V*_Total_ and *D*_A_ were denoted as specific surface area, total pore volume and mean pore sizes, respectively.

The FT-IR and Raman spectra of MoS_2_-IS and FeMoS_2_-IS showed that for the MoS_2_-IS, five characteristic peaks appeared, located at 1619, 1420, 1253, 804, and 582 cm^−1^, in the region of 2000–500 cm^−1^, which belonged to the characteristic peaks of Mo–S (Fig. 1b and c).^34,35^ After treatment, similar characteristic peaks appeared at the above positions, implying that Mo–S structure in the catalyst remained unchanged. Nevertheless, it was difficult to identify structural differences between them. Therefore, they were characterized by Raman analysis. MoS_2_-IS exhibited some characteristic peaks located at 445–448, 402, and 376 cm^−1^, which were attributed to the structure of 2H-MoS_2_.^35^ In addition, some characteristic peaks located at 336, 283, and 150 cm^−1^ were observed, belonging to the structure of 1T-MoS_2_.^35^ The results demonstrated the co-existence of 2H–1T MoS_2_ structure in MoS_2_-IS. The characteristic peaks located at 993, 819, and 664 cm^−1^ were observed, which were ascribed to the structure of MoO_3_.^35^ Thus, MoS_2_-IS was concluded to be composed of MoS_2_ and MoO_3_. After modification, the above characteristic peaks basically disappeared and some new characteristic peaks located at 969, 777, 348, 280, and 214 cm^−1^ appeared. This suggested that a new structure in FeMoS_2_-IS was formed, which was different from MoS_2_ and MoO_3_.

**Fig. 2:**
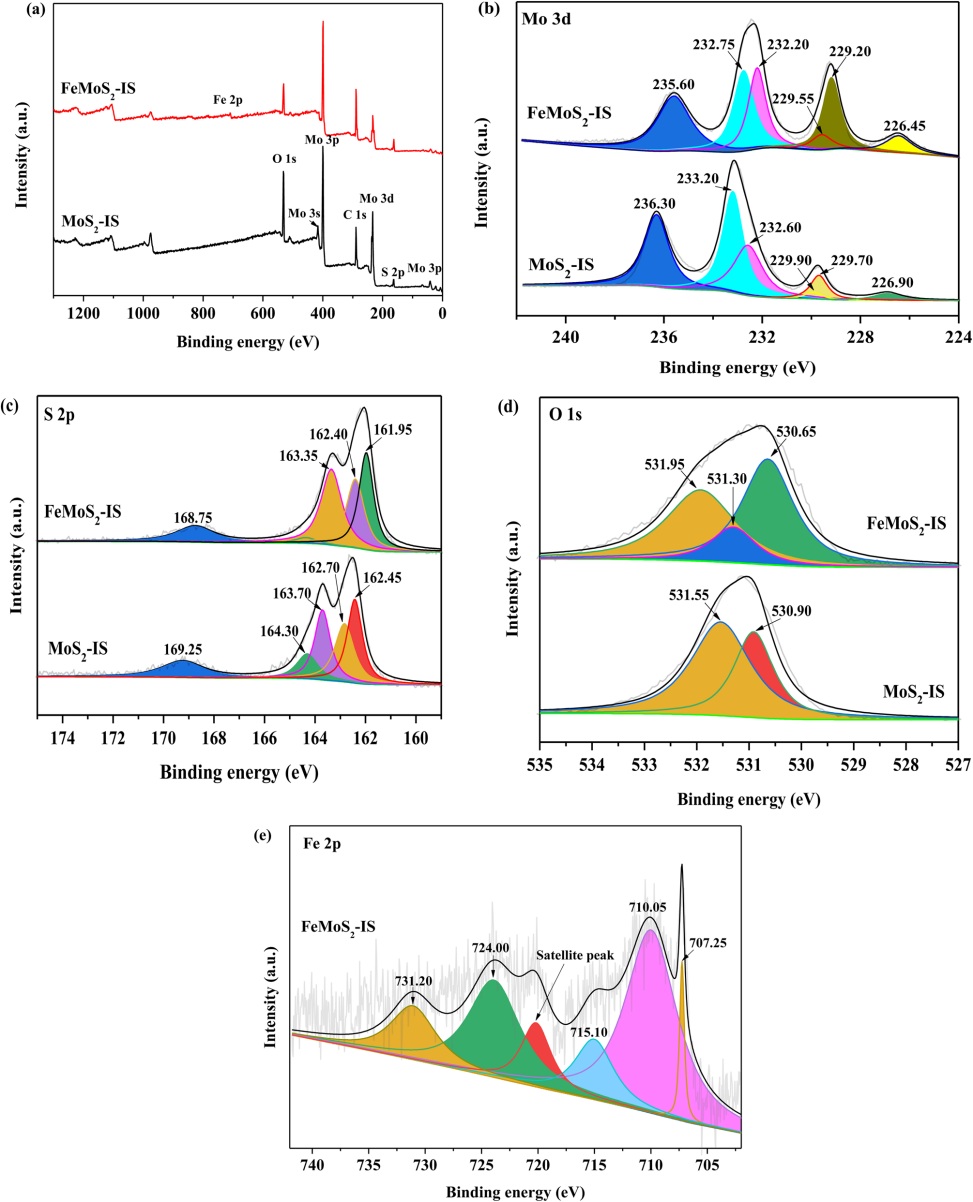
XPS spectra of (a) survey spectra, (b) Mo 3d, (c) S 2p and (d) O 1s in the MoS_2_-IS and FeMoS_2_-IS, and (e) Fe 2p in the FeMoS_2_-IS.

The surface elemental information of MoS_2_-IS and FeMoS_2_-IS was confirmed by XPS survey spectra, with the synthesized MoS_2_-IS and FeMoS_2_-IS primarily composed of Mo, S, and O (Fig. 2a). In addition, Fe also appeared in FeMoS_2_-IS. In terms of Mo-3d of MoS_2_-IS, one weak binding energy peak was located at 226.90 eV, which was attributed to S atoms at the external side of Mo–S (Fig. 2b).^34–36^ There were also two binding energy (BE) peaks located at 229.75 and 233.05 eV, which respectively corresponded to characteristic peaks of Mo 3d_5/2_ and Mo 3d_3/2_, which indicated that Mo^4+^ was present in MoS_2_-IS.^36^ The characteristic BE peak located at 229.75 eV was subdivided into two characteristic peaks located at 229.90 and 229.69 eV, corresponding to the structure of 2H-MoS_2_ 3d_5/2_ and 1T-MoS_2_ 3d_5/2_.^35^ Similarly, the characteristic BE peak located at 232.40 eV was subdivided into two characteristic peaks with the BEs at 233.20 and 232.57 eV, corresponding to the structure of 2H–MoS_2_ 3d_3/2_ and 1T-MoS_2_ 3d_3/2_,^35^ respectively. The above results showed that a mixed structure of 2H- and 1T-MoS_2_ existed in MoS_2_-IS. In addition, one clear characteristic BE peak located at 236.30 eV corresponded to a characteristic peak of Mo-based oxides.^34–36^

After treatment, the characteristic peak of Mo 3d was altered. The ratio of Mo^4+^ to Mo^6+^ and S atoms from the external of Mo–S were both increased, but the ratio of 2H- and 1T-MoS_2_ and concentration of Mo^6+^ were both reduced in FeMoS_2_-IS, compared to MoS_2_-IS (Table S1†). This meant that this modification contributed more Mo^4+^ and external S in Mo–S and/or FeS_2_. As mentioned above, the decomposition of PMS could be accelerated by enhancing the Fe^2+^ and Fe^3+^ cycle in the presence of Mo^4+^.^13–18^ Simultaneously, more external S atoms in the catalyst were in better contact H^+^ in solution such that more active sites, such as Fe^2+^ and Mo^4+^, were exposed. Some authors have pointed out that Mo^4+^, not Mo^6+^, species were important active sites for decomposing PMS.^34–36^ Accordingly, more Mo^4+^ and less Mo^6+^ species were in favor of strengthening PMS activation. For S 2p in MoS_2_-IS, there were two distinct characteristic BE peaks located at 163.70 and 162.65 eV,^19,35^ which was respectively ascribed to the S 2p_1/2_ and S 2p_3/2_ (Fig. 2c). In addition, one characteristic peak located at 169.24 eV was attributed to –SO_*n*_– due to oxidation during catalyst synthesis.^35^ After modification, the characteristic peak of S 2p was little changed. Interestingly, the O 1s spectrum could be deconvoluted into two components located at 531.54 and 530.91 eV, corresponding to hydroxyl groups in adsorbed water and O defects in the MoS_2_-IS (Fig. 2d). After modification, besides the above peaks, there appeared a new peak located at 531.30 eV, which was ascribed to surface adsorbed O species.^35^ In the case of Fe 2*p* in FeMoS_2_-IS (Fig. 2e), a peak located at 707.25 eV was attributed to the existence of FeS_2_.^32^ Also, the extra three characteristic peaks, located at 709.99 and 731.07 eV, corresponded to Fe^2+^.^37^ Besides, the peak located at 720.18 eV was attributed to the satellite peak. This indicated that Fe^2+^ was generated in the FeMoS_2_-IS due to the reduction of Fe^3+^ by thiourea. There were two characteristic peaks located at 715.02 and 723.90 eV, which corresponded to Fe^3+^. According to the peak areas of Fe^2+^ and Fe^3+^, the Fe^2+^ concentration and Fe^2+^/Fe^3+^ ratio were 0.67 and 1.99, respectively, suggesting that most Fe^3+^ in ferric nitrate was reduced to Fe^2+^ during synthesis. The higher Mo^4+^/Mo^6+^ and a large number of Fe^2+^ species were concluded to have been generated after Fe modification.

### 3.2. Removal of organic pollutants in different catalytic reaction processes

**Fig. 3:**
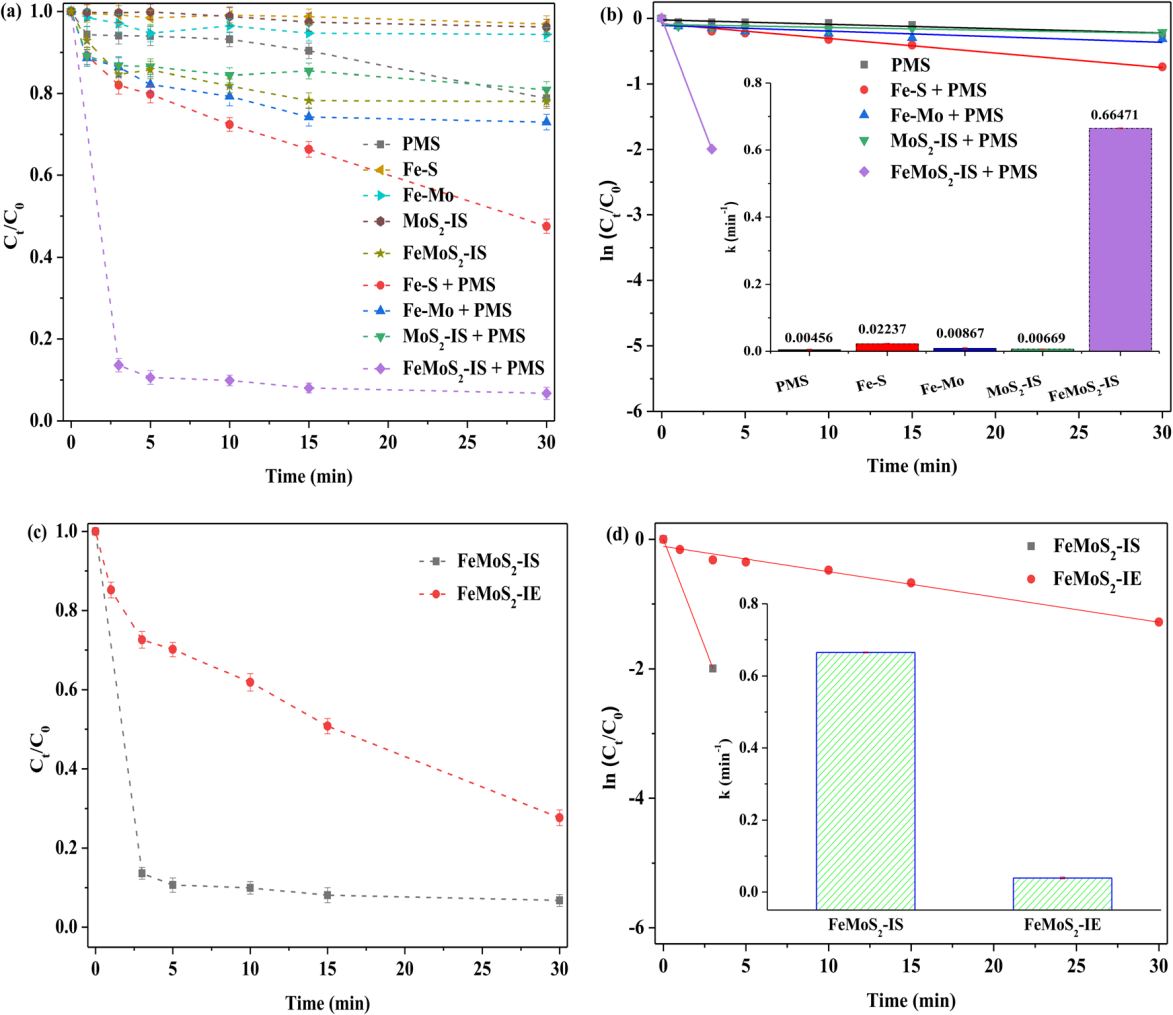
(a) RhB degradation of adsorption and different PMS system; (b) pseudo first order kinetics of different catalytic system and reaction rate constant (illustration); (c) removal of RhB activating PMS by FeMoS_2_ with different methods; (d) pseudo first order kinetics of different FeMoS_2_ activating PMS system and reaction rate constant (illustration). Reaction conditions: [RhB] = 10 mg L^−1^, initial pH = 3.0, [Catal. ] = 1.0 g L^−1^, [PMS] = 1.0 mM and in the darkness.

Different catalysts for activating PMS in RhB removal in the darkness were compared. The RhB degradation efficiency was only 21% when PMS was used alone (Fig. 3). Although MoS_2_-IS was added into the above system, RhB removal hardly increased. According to the pseudo-first order reaction equation, the reaction rate constant (denoted as *k*) was calculated.^20^ These values were 0.00456 and 0.00669 min^−1^ for PMS and PMS + MoS_2_-IS, meaning that their reaction rates were extremely slow. This was because the MoS_2_-IS contained a lot of Mo^6+^ species, such as MoO_3_, as evidenced from XPS characterization, but it did not act as active sites.^19^ Also, its low *S*_BET_ restricted increased catalyst activity and, accordingly, RhB removal was poor. The degradation efficiency of RhB was ∼27% at 0.00867 min^−1^ for its *k* using Fe–Mo as a heterogeneous catalyst. This indicated that the bimetallic Fe–Mo catalyst containing no S could activate PMS to remove RhB, which was in agreement with the literature.^38^ However, this promotion was limited and also illustrated that the S was very important factor for improving RhB removal. If a catalyst without Mo was employed, a positive effect should be achieved. Surprisingly, the results showed that the RhB degradation efficiency over Fe–S was only ∼52%, with *k* at 0.02237 min^−1^. This was only due to the generation of FeS_2_. Clearly, the role of Mo was important in the removal of RhB. In contrast, when FeMoS_2_-IS was used as a heterogeneous catalyst under identical reaction conditions, ∼86% of RhB was removed, with *k* at 0.66471 min^−1^, obtained only in 3.0 min in the presence of PMS. From the above results, FeMoS_2_-IS possessed many active sites such as FeS_2_ and S vacancies and their synergism activated PMS to form ROS. Zhou *et al.*^26^ have directly used pyrite for activating PMS for the effective degradation and mineralization of diethyl phthalate. Huang *et al.*^39^ have considered that S vacancies accelerate electron transfer and reduce Mo^6+^ to Mo^4+^ in natural molybdenite/PMS systems. As a result, RhB removal was significantly increased relative to the control experiment.

As mentioned earlier, Fe-doped MoS_2_ was mainly synthesized *via* a post treatment.^20–22^ Herein, FeMoS_2_-IE was obtained by impregnation and high-temperature successive treatments and then it was applied for activating PMS. The results indicated that the RhB degradation efficiency, with *k* at 0.03914 min^−1^, reached ∼72% at 30 min. The diffraction characteristic peaks of FeMoS_2_-IE were mainly ascribed to the mixed structure of MoS_2_ and MoO_3_ (Fig. S4†). Meanwhile, Fe species were not detected because they were either highly dispersed on the support or in low crystallinity. On the basis of FeMoS_2_-IS and FeMoS_2_-IE, a great difference was found on RhB removal, which was due to different structures between them. As mentioned above, the main phases in the former were MoS_2_ and MoO_3_ whereas the dominant phases in the latter were FeS_2_. As shown in eqn (2)–(5),^32,33^ the FeS_2_ to generate dissolved as well as surface Fe^2+^ was an important reason for promoting RhB degradation. In a few words, this provided a new method for FeS_2_ synthesis. In addition, this had many merits, *e.g*., simple operation, low cost, and green. Accordingly, it possessed great potential for industrial application.

2 FeS_2_ + H^+^ → Fe^2+^ + H_2_S

3 FeS_2_ + 3.5O_2_ + 4H_2_O → Fe^2+^ + 2SO_4_^2−^ + 2H^+^

4 2FeS_2_ + 7O_2_ + 2H_2_O → 2Fe^2+^ + 4SO_4_^2−^ + 4H^+^

5 FeS_2_ + 14Fe^3+^ + 8H_2_O → 15Fe^2+^ + 2SO_4_^2−^ + 16H^+^

**Fig. 4:**
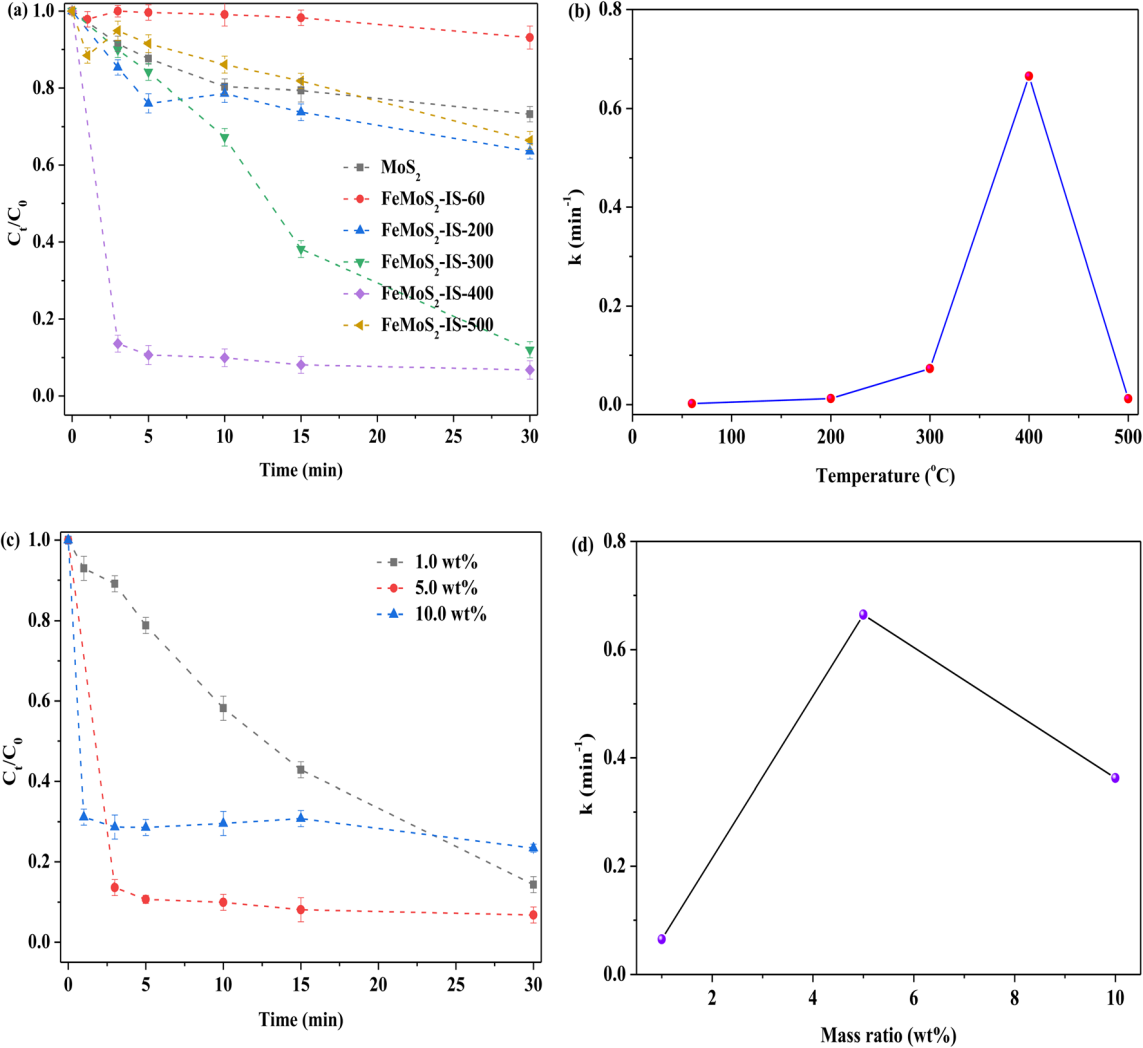
(a) Effect of the calcined temperatures of catalyst on the removal of RhB; (b) relationship between calcined temperatures and reaction rate constant; (c) effect of the Fe concentration on the removal of RhB; (d) relationship between Fe concentration and reaction rate constant. Reaction conditions: [RhB] = 10 mg L^−1^, initial pH = 3.0, [Catal. ] = 1.0 g L^−1^, [PMS] = 1.0 mM and in the darkness.

To further explore the roles of FeS_2_ and S vacancies, the treatment temperature and Fe doping were investigated, particularly how temperature affected the formation of FeS_2_ and S vacancies (Fig. 4a). When the treatment temperature was 400 °C, RhB removal was the highest, while with further elevation of temperature, RhB removal was clearly reduced. When the catalyst was not heat treatment, besides there being no FeS_2_ and Mo–S, there was lower Mo^4+^/Mo^6+^ and Fe^2+^/Fe^3+^ ratios, and higher concentrations of Mo^6+^ and 2H-/1T-MoS_2_ obtained in FeMoS_2_-IS-60 relative to FeMoS_2_-IS-400 (or FeMoS_2_-IS), as evidenced by XRD and XPS results (Fig. S5 and S6a–S6e and Table S1†). Accordingly, RhB removal was low. As the temperature was increased, S in the catalyst reacted with atmospheric O to form gaseous SO_2_ and thus S vacancies generated and more Fe^2+^ and Mo^4+^ species exposed. In addition to the activation of PMS by Fe^2+^ and Mo^4+^, S vacancies were able to activate PMS to form ROS.^19,29^ This was due to the ability of defective sites to reduce the adsorption energy of PMS and prolong the bond length of peroxides (–O–O–) in PMS. As the indirect evidence, the comparison of MoS_2_ and MoS_2_-300 for activating PMS on RhB removal was conducted. RhB removal over MoS_2_-300 was remarkably higher than that of MoS_2_ (Fig. S7†). This result suggested that S vacancies could indeed facilitate RhB removal. Also, more FeS_2_ was generated with increased calcination temperature and, as a result, RhB removal increased. When the temperature was too high, FeMoS_2_-IS structure was seriously destroyed, as evidenced from TG-DSC characterization (Fig. S8†). As a consequence, RhB degradation was remarkably reduced.

As mentioned above, Fe modification was in favor of strengthening RhB removal. On the basis of this reason, the influence of Fe concentration was examined in FeMoS_2_-IS. When the catalyst Fe concentration was increased from 1.0 to 5.0 wt%, RhB degradation efficiency became rapid, at ∼86% in 3.0 min, as compared to that of the former at 30 min (Fig. 4c and d). Further increasing Fe concentration, RhB removal was remarkably reduced. XRD results showed that the main phase was not FeS_2_ in the 1.0 and 10.0 wt% Fe-modified catalysts (Fig. S9†). Therefore, RhB removal was decreased. In brief, the above results well illustrated that FeS_2_ and S vacancies were two essential factors for enhancing RhB degradation.

### 3.3. Effects of operation parameters on RhB removal

**Fig. 5:**
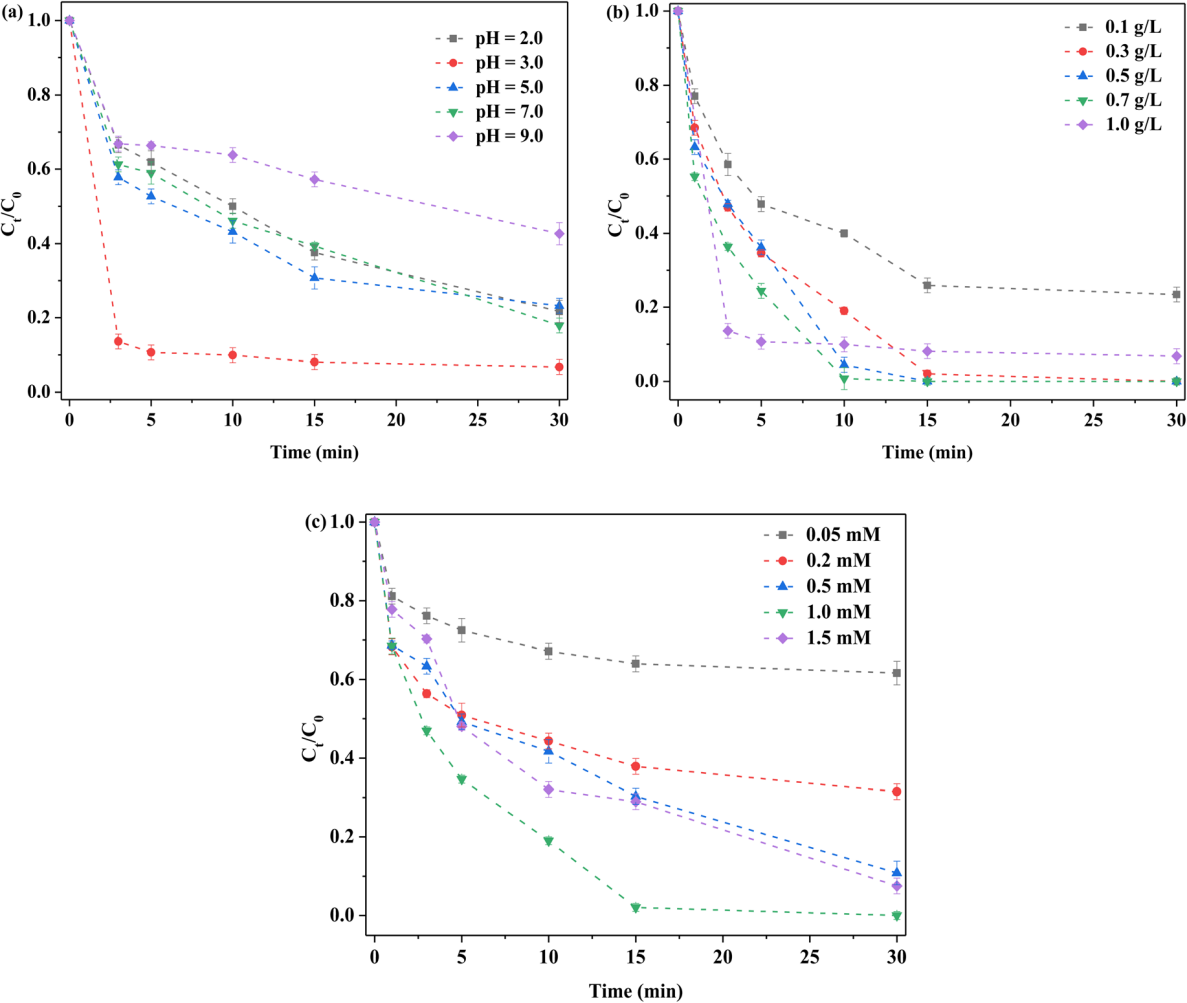
Optimized reaction conditions on the removal of 10 mg L^−1^ RhB in the FeMoS_2_-IS catalyzed PMS system in the darkness. (a) Initial pH. Reaction conditions: [FeMoS_2_-IS] = 1.0 g L^−1^ and [PMS] = 1.0 mM; (b) catalyst dosage. Reaction conditions: initial pH = 3.0 and [PMS] = 1.0 mM; (c) PMS concentration. Reaction conditions: initial pH = 3.0 and [FeMoS_2_-IS] = 0.3 g L^−1^.

Examination of the effects of initial pH on RhB removal revealed that removal was more favorable under acidic conditions, compared to neutral and alkaline conditions (Fig. 5a). When the initial pH was 3.0, RhB removal was the highest, while at pH 7.0, the obtained result was still satisfactory. Removal was decreased at pH 9.0, at ∼50%. Under an acidic environment, S atoms on the surface of FeMoS_2_-IS were trapped by H^+^ in solution to generate H_2_S,^22,23^ in which case, more active sites, such as Fe^2+^ and Mo^4+^, were exposed and thus more ROS generated, such that the above co-actions promoted RhB removal. In an alkaline environment, this effect was suppressed due to inhibition of S atom capture and, accordingly, RhB degradation decreased. In addition, after PMS was added into the reaction system, the solution became acidity. Except for an initial pH of 2.0, all solutions retained about pH 3.0 after reaction (Fig. S10†). In this case, the above-mentioned effect was to a lesser extent inhibited. The zeta potential value of FeMoS_2_-IS was −1.66 mV at ∼3.0 pH while the RhB was a cationic dye. Thus, this was in favor of adsorbing RhB. Generally, RhB removal was high over a wide pH range.

Based on the above results, the effects of FeMoS_2_-IS dosages were examined (Fig. 5b). When 0.1 g L^−1^ of catalyst was applied, RhB degradation efficiency was ∼77% in 30 min. With increased dosage, the degradation rate was accelerated. When the catalyst dosage was within 0.3–0.7 g L^−1^, all degradation efficiency was ∼100% in 15 min. However, when the dosage exceeded 0.7 g L^−1^, the removal started to decrease. The dosage of catalyst was insufficient, as there was not enough active sites to activate PMS. Therefore, the removal of RhB was low. With increasing catalyst dosage, more ROS were produced and, hence, the removal increased. When the catalyst dosage was too high, agglomeration occurred between catalyst particles, thereby affecting PMS activation. Meanwhile, the repulsion force between catalyst and PMS increased due to their similar charges.^40^ Similarly, the removal was decreased.

Here, the PMS concentration was optimized. The degradation efficiency of RhB was ∼38% at 30 min when 0.05 mM PMS was employed (Fig. 5c). With increased PMS concentration, RhB removal increased. When the PMS concentration was 1.0 mM, the removal reached the highest and began to decrease with further increased PMS. Increased PMS concentration generated more ROS and thus produced higher RhB removal. When PMS was too high, the removal decreased, which was mainly due to the reaction between partially activated PMS without organic pollutants. In addition, there was newly formed ROS, such as HO_2_˙^−^.^41^ The activity of these species was less than that of the original system. Accordingly, RhB degradation was reduced. In short, optimal reaction conditions were established and therefore the best degradation obtained.

**Fig. 6:**
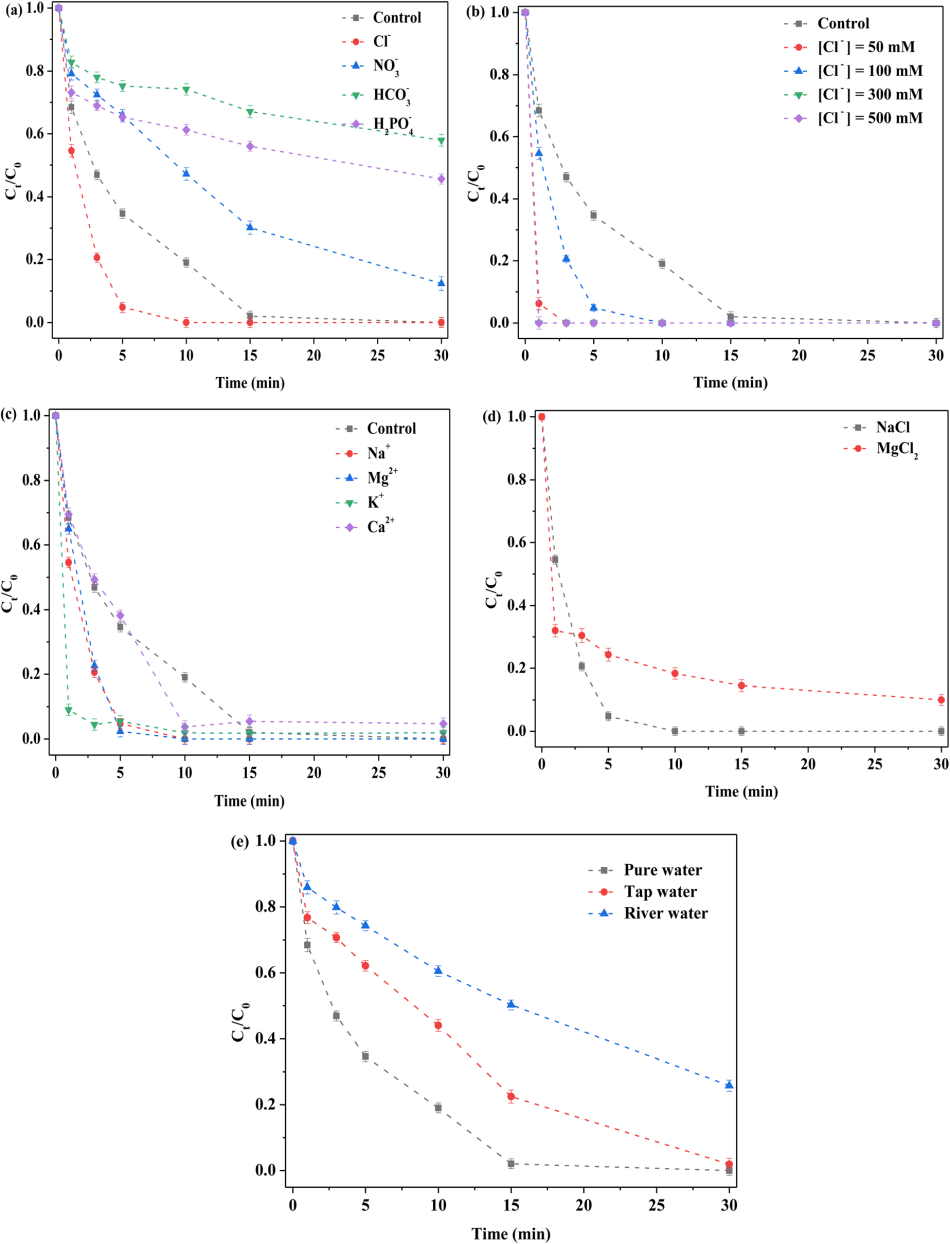
Effect of various factors on the removal of RhB. (a) Anions; (b) Cl^−^ concentration; (c) cations; (d) different cations with the same concentration of Cl^−^; (e) types of water. Reaction conditions: [RhB] = 10 mg L^−1^, initial pH = 3.0, [FeMoS_2_-IS] = 0.3 g L^−1^, [PMS] = 1.0 mM, [anions or cations] = 100 mM and in the darkness.

Common anions, including chloride, nitrate and bicarbonate ions (Cl^−^, NO_3_^−^, and HCO_3_^−^, respectively) were captured by the highly oxidizing free radical species and the corresponding reaction rate constants considerably varied.^22,23,42–44^ In this reaction system, different cations and anions were respectively added. RhB degradation efficiency was observed to be close to 100% after 15 min of reaction without any extra anions (Fig. 6a). When different anions (Cl^−^, NO_3_^−^, HCO_3_^−^, and H_2_PO_4_^−^) were employed, great changes appeared in RhB degradation. Cl^−^ promoted removal based on the original reaction system, whereas other anions (including NO_3_^−^, HCO_3_^−^, and H_2_PO_4_^−^) suppressed degradation. After adding Cl^−^, the degradation efficiency reached 100% in 10 min, which indicated that Cl^−^ played a positive role in RhB removal. To further explore this effect, different concentrations (50, 100, 300 and 500 mM) of NaCl were investigated (Fig. 6b). RhB removal ability was observed to be clearly strengthened by introducing low or high NaCl concentrations, which further confirmed that Cl^−^ was indeed a beneficial additive. This was because Cl^−^ could react with strong free radical species to form other free radical species.^41^ Accordingly, the promotion might have been attributable to newly formed active species such as Cl˙ and Cl_2_˙. In addition, other active chlorine species were formed.^42,43^ For NO_3_^−^, RhB removal decreased, which might have been attributable to the competition for reactants. For the HCO_3_^−^, the degradation was severely inhibited after HCO_3_^−^ addition, because solution pH was altered and sharply increased toward 8–9. Under alkaline condition, the binding of H^+^ with S atoms on the catalyst surface did not occur,^22,23^ thereby reducing the exposure of active sites, such as Mo^4+^ and Fe^2+^ species. In addition, HCO_3_^−^ reacted with PMS or its related ROS.^20^ Thus, removal was clearly decreased after HCO_3_^−^ was added. Similar to HCO_3_^−^, the solution pH was remarkably increased after H_2_PO_4_^−^ was added to the reaction system, such that it was clear that an inhibition effect occurred due to alkaline condition. In addition, interactions between H_2_PO_4_^−^ and Mo on the catalyst could generate a heteropoly acid salt.^45^ Hence, H_2_PO_4_^−^ had a clear negative influence on RhB removal.

To explore the influence of cations on RhB removal, various cations, including Na^+^, K^+^, Mg^2+^, and Ca^2+^ with Cl^−^ as the anion were examined in detail. The order of the activity in terms of RhB removal was Na^+^ > Mg^2+^ > K^+^ > Ca^2+^ > control (Fig. 6c). The results indicated that these cations had no inhibitory effect on RhB degradation. Promotion might have been due to the presence of Cl^−^. To explore the contribution of Na^+^ and Cl^−^, Na^+^ was replaced by Mg^2+^ with the same concentration of Cl^−^ (Fig. 6d). When MgCl_2_ was used as the precursor, RhB removal was significantly lower than that of NaCl as precursor. Thus, the above- mentioned promotion was attributed to the coaction of cations and anions, with the contribution of Cl^−^ greater than that of Na^+^. To explore the influence of water types, tap water and river water (Xiang-Jiang River, Hunan, China) as solvents were selected. The RhB degradation efficiencies were 100 and 70% in tap and river water at 30 min, respectively (Fig. 6e). In general, the catalyst exhibited satisfactory degradation in different water environments. Using river water as solvent, the low degradation might have been due to the presence of natural organic humic acids and various inorganic salts. In short, the FeMoS_2_-IS/PMS system had a strong ability to resist external interference. In addition, different organic pollutants were investigated in this system. Most of organic pollutants (RhB, MB, MO, and AOII) were rapidly removed in 30 min, especially AOII (Fig. S11†). These results indicated that the catalytic system possessed excellent ability to remove organic pollutants in wastewater.

The stability of FeMoS_2_-IS was examined and it was observed that the degradation efficiency of RhB reached 100% with its 1st cycle of reaction (Fig. S12†). After reaction, the catalyst was regenerated by filtering the reaction solution, washing the solid with distilled water and ethanol to separate liquid and solid, and dried. Finally, the dried sample was calcined at 400 °C for 1.5 h. According to the same reaction conditions as the 1st reaction, the regenerated catalyst was added into a 2nd reaction solution. The results indicated that the degradation efficiency of RhB arrived at 100% after 30 min of reaction. Repeating the above same steps, several reactions and regeneration were conducted. After the 5th reaction, the degradation efficiency of RhB was slightly decreased, implying that the catalyst possessed basically retained original activity. Notably, the used catalyst had to be treated at high temperature to recover the initial catalytic activity because it was covered and/or blocked the active sites of catalyst by the intermediates and/or products, which was the main reason for remarkably decreased RhB removal (Fig. S13†). By a simple calcination, the used catalyst was well regenerated.

The structure of fresh and used FeMoS_2_-IS was characterized by XRD, which showed that used catalyst was greatly changed after the several reaction cycles, compared to that of fresh catalyst, with the diffraction characteristic peaks of FeS_2_ no longer apparent, which was mainly attributed to the structure of MoO_3_ (Fig. S14†). This situation mainly originated from the leaching of Fe and oxidation of MoS_2_ during reaction and regeneration, which were the main reasons for decreasing catalytic activity after the reaction and regeneration. To further confirm this difference, the changes in surface elements on the catalyst after reaction were characterized by XPS. One characteristic peak located at ∼707.25 eV did not appear in Fe 2p, implying that Fe–S had disappeared and, meanwhile, the ratio of Fe^2+^ and Fe^3+^ was remarkably reduced, meaning that a lot of Fe^2+^ were oxidized to Fe^3+^ (Fig. S15 and Table S1†). This change was further confirmed in S 2p, where the S^2−^ was completely eliminated and the peak was attributed to one characteristic of SO_*n*_^−^. This indicated that Mo–S and Fe–S on the catalyst surface did not indeed exist. For Mo 3d, the ratio of Mo^4+^ and Mo^6+^ was reduced whereas the concentration of Mo^6+^ increased, hinting that great oxidation was occurred. This was confirmed by O 1s XPS characterization; that is, the concentration of lattice O in the catalyst had increased, which was further supported by the fact that the color of the catalyst had turned from black to white. The above results illustrated that the heterogeneous reaction was happened on the surface of catalyst.

### 3.4. Leaching of various ions during reaction and their effects

**Fig. 7:**
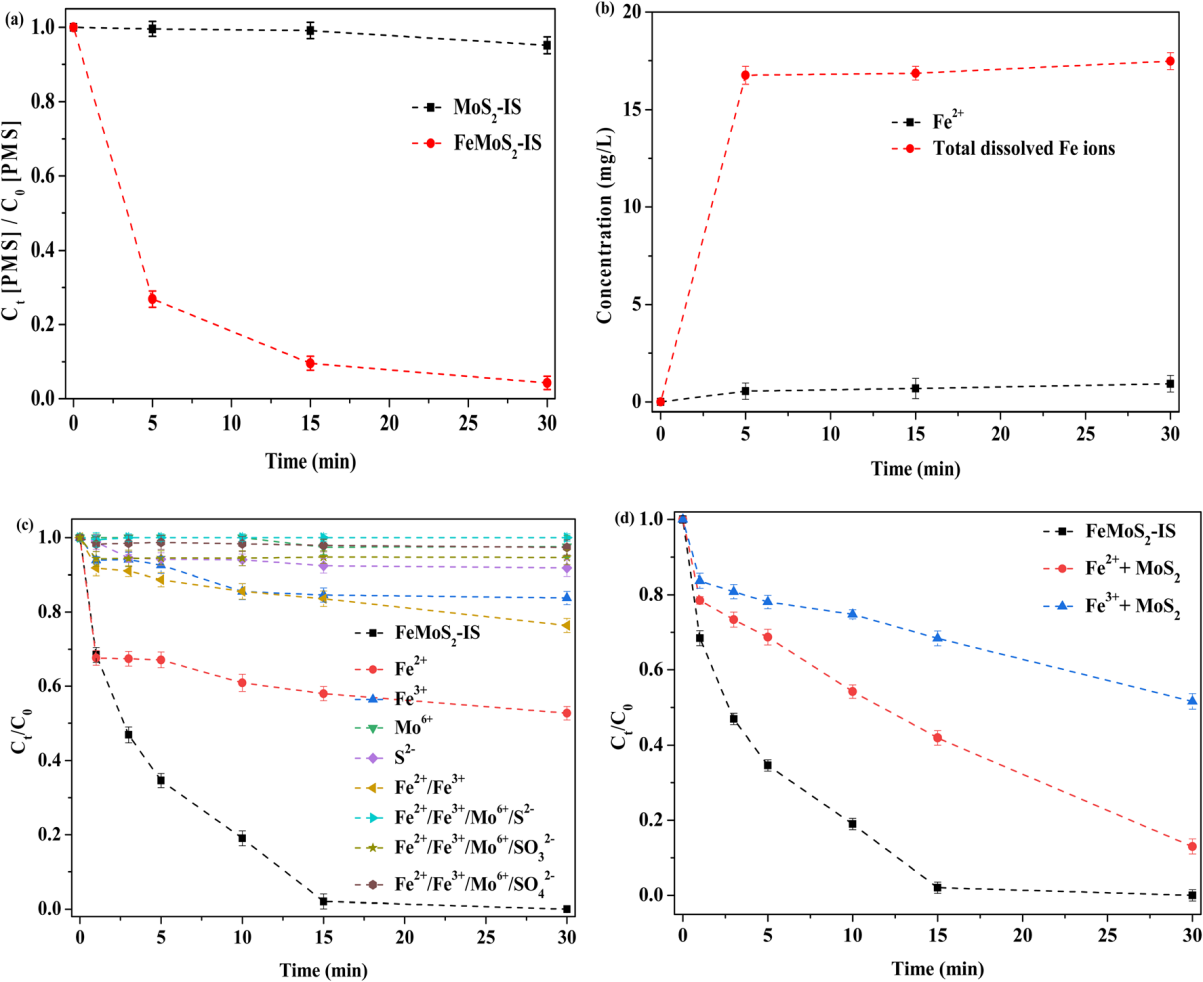
(a) The decomposition of PMS; (b) the leaching of Fe^2+^ and total dissolved Fe ions; (c) removal of RhB in the PMS-based homogeneous reaction system; (d) removal of RhB in the PMS-based heterogeneous reaction system. Reaction conditions: [RhB] = 10 mg L^−1^, initial pH = 3.0, [FeMoS_2_-IS and MoS_2_] = 0.3 g L^−1^, [PMS] = 1.0 mM, [Fe^2+^] = 0.55 mg L^−1^, [Fe^3+^] = 15.46 mg L^−1^, [Mo^6+^] = 1.11 mM, [S^2−^] = [SO_3_^2−^] = [SO_4_^2−^] = 0.14 mM and in the darkness.

Decomposition of PMS had an important influence on ROS formation and the test was conducted.^30^ When MoS_2_-IS acted as catalyst, the decomposition efficiency of PMS was no more than 5% at 30 min (Fig. 7a). This was main reason for the low RhB degradation. In contrast, when FeMoS_2_-IS was the catalyst, the decomposition efficiency of PMS reached as high as ∼95% in the same time. Thus, FeMoS_2_-IS had a higher ability to activate PMS and thus removed more RhB in solution. Also, the source of ROS was mainly from PMS decomposition.

During reaction, some ions were leached. To examine this, Fe^2+^ and total Fe ions were evaluated (Fig. 7b). After reaction to 30 min, the concentrations of Fe^2+^ and total Fe ions were 0.55 and 16.01 mg L^−1^, respectively. Based on the above eqn (2)–(5), soluble Fe ions were generated. According to the calculation, the loss efficiency of total Fe ions was ∼5.0 wt%, implying that the catalyst had a good stability. Similarly, Mo and S ions moved from the catalyst and entered the reaction solution. Thus, the effect of their activation on PMS in the homogeneous system was investigated (Fig. 7c). When Fe^2+^ alone was used as homogeneous catalyst, the degradation efficiency of RhB was 47% after 30 min of reaction, which suggested that dissolved Fe^2+^ made some contributions on RhB removal because Fe^2+^ itself could directly activate PMS.^14–16^ The degradation efficiency of RhB was both below 25% when Fe^3+^ or Fe^2+^/Fe^3+^ were employed, which indicated that these Fe ions' ability to activate PMS was poor. When soluble Mo ion ((NH_4_)_6_Mo_7_O_24_·H_2_O as precursor) was used, the degradation efficiency of RhB was only ∼2% at 30 min, hinting that dissolved Mo ion had little activation effect on PMS. Similarly, S^2−^ (Na_2_S as a precursor) alone could not accelerate the removal rate. Surprisingly, the removal did hardly occur when all dissolved ions (Na_2_SO_3_ and Na_2_SO_4_ as SO_3_^2−^ and SO_4_^2−^ precursors, respectively) were simultaneously added into the reaction system. This result indicated that it contributed the extremely low degradation for RhB even when various ions from the catalyst leached during reaction.

It has been reported that the homogeneous Fe^2+^ or Fe^3+^ ions can remarkably enhance RhB removal in the MoS_2_/PMS system.^14–18^ To verify this deduction, a series of experiments were designed. The degradation efficiency of RhB was respectively 87 and 48% in Fe^2+^/MoS_2_/PMS and Fe^3+^/MoS_2_/PMS systems, which meant that Fe ions dissolved by interactions with MoS_2_ in the FeMoS_2_-IS could accelerate RhB removal (Fig. 7d). In other words, the heterogeneous catalytic reaction played a vital role on RhB removal. To date, there has been a debate regarding the main active sites in the catalyst FeS_2_ activating persulfate. Some researchers^46,47^ have suggested that Fe^2+^ played a major role. Others^48^ have pointed out that S species were involved in this reaction in addition to Fe^2+^. Exploring whether Fe^2+^ was on the catalyst surface or in solution, different researchers have come to different conclusions: (i) dissolved Fe^2+^;^49^ (ii) Fe^2+^ on the surface of heterogeneous catalyst;^50^ and (iii) Fe^2+^ in the both solution and heterogeneous catalysts.^51^ Clearly, this reaction behavior was different from that of FeS_2_ catalytic PMS systems in previous studies.

### 3.5. Identifying ROS in RhB removal

**Fig. 8:**
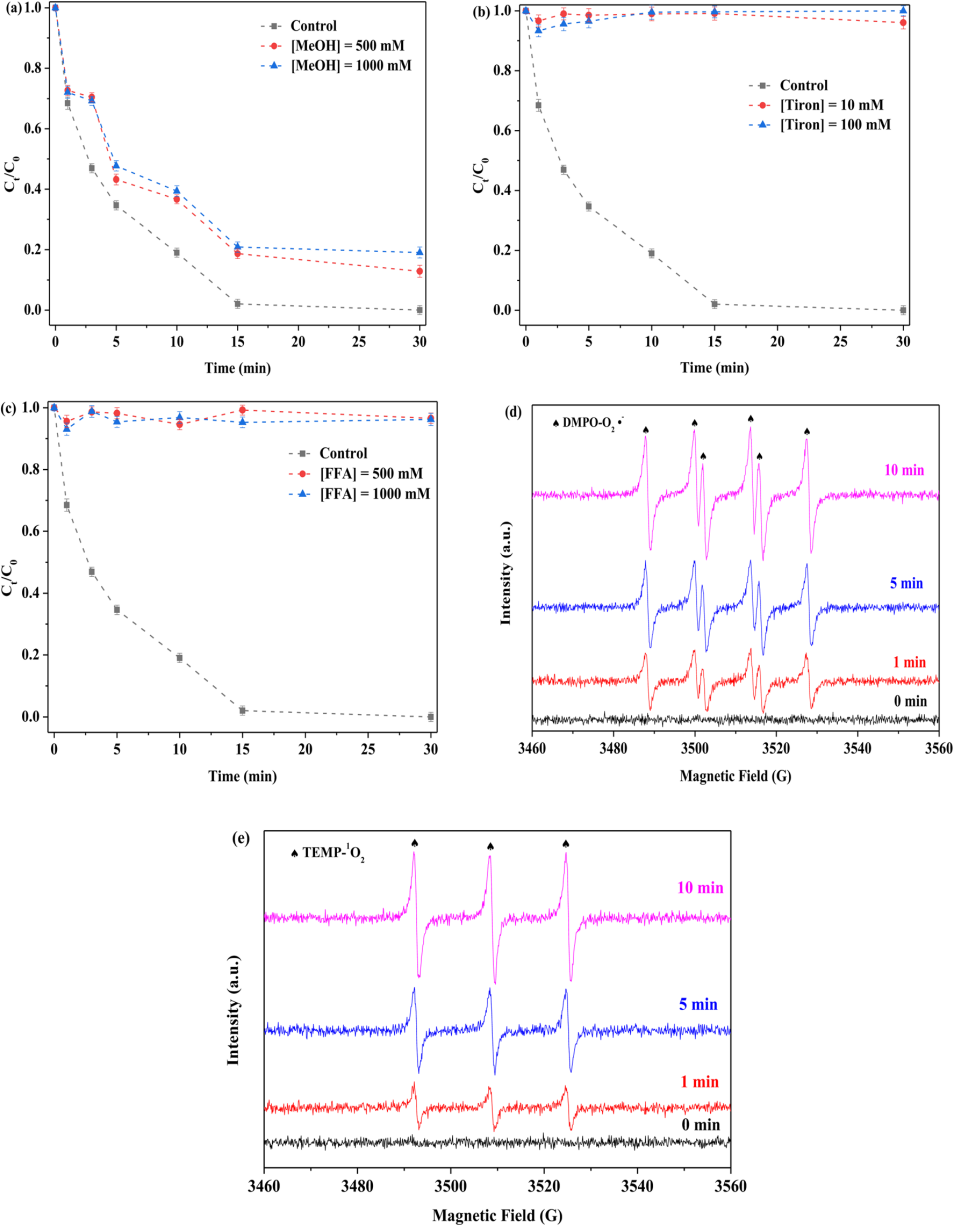
Effect of (a) MeOH, (b) Tiron and (c) FFA on the removal of RhB in the FeMoS_2_-IS/PMS process and signals of EPR (d) and (e). Reaction conditions: [RhB] = 10 mg L^−1^, initial pH = 3.0, [FeMoS_2_-IS] = 0.3 g L^−1^, [PMS] = 1.0 mM and in the darkness.

To examine the mechanism of PMS activation, different quenchers were used to detect possible ROS in the FeMoS_2_-IS/PMS system. In general, free radical species (including SO_4_˙^−^, ˙OH, and O_2_˙^−^) and non-free radical species, such as singlet oxygen (^1^O_2_), usually appeared in the process of PMS activation. In this experiment, methanol (MeOH) was used to identify as SO_4_˙^−^ and ˙OH,^13–15^ tiron applied for capturing O_2_˙^−^,^52,53^ and furfuryl alcohol (FFA) employed for capturing ^1^O_2_,^14^ as shown in Fig. 8a–c. FeMoS_2_-IS was an Fe-based catalyst in the Fenton-like reaction and SO_4_˙^−^ and ˙OH usually produced during PMS activation. This was verified by the reaction with MeOH. After MeOH in different concentrations (500 and 1000 mM) was employed, RhB degradation efficiency decreased from 100 to 89% and 85% in 30 min, respectively, implying the presence of SO_4_˙^−^ and ˙OH in the reaction solution but with limited effect. After the addition of phenol with different concentrations, the inhibitions were clearly observed (Fig. S16(a)†), implying that the above two species were appeared on the surface of catalyst. It was further confirmed by the EPR characterization. As shown in Fig. S16(b),† the signals of SO_4_˙^−^ and ˙OH were appeared and the signal of ˙OH turned stronger with increasing reaction time, implying that the ˙OH was generated from the reaction between SO_4_˙^−^ and H_2_O.^15^ This was mainly attributed to the activation of PMS by Fe^2+^ and Mo^4+^.^13–23^ The above results confirmed that the SO_4_˙^−^ and ˙OH were not main ROS in this reaction. When tiron at different concentrations (10 and 100 mM) was used, RhB degradation efficiencies were both <5% in 30 min. This indicated that the reaction system contained a lot of O_2_˙^−^ species and played an important role in degradation. When FFA at different concentrations (500 and 1000 mM) was applied, RhB degradation efficiencies were both no more than 5%, which showed that ^1^O_2_ existed in the reaction system. Notably, FFA could react with PMS, resulting in reduced RhB removal.^18^ To confirm the existence of O_2_˙^−^ and ^1^O_2_ in the reaction system, EPR characterization was applied using DMPO (methanol as solvent) and TEMP, respectively. Some noticeable signals of DMPO-O_2_˙^−^ adduct were detected in the FeMoS_2_-IS/PMS system, confirming that the appearance of O_2_˙^−^ had occurred (Fig. 8d). Similarly, remarkable signals of TEMP-^1^O_2_ were observed, implying that many ^1^O_2_ were formed in this system (Fig. 8e). This reaction was concluded to indeed contain a mixture of radical and non-radical species, with O_2_˙^−^ and ^1^O_2_ as main ROS.

### 3.6. Feasible synthetic and reaction mechanisms

When all the precursors were added into the water, the solution pH was 1.8. In this environment, the Fe and Mo ions existed in the form of Fe^3+^ and Mo_7_O_24_^6−^. After thiourea introduction, it combined with Fe^3+^ and Mo_7_O_24_^6−^. Under hydrothermal condition, some Fe^3+^ and Mo^6+^ were reduced to Fe^2+^ and Mo^4+^ by thiourea to form the polymer structure, namely, –Mo–S_2_–Fe–S_2_–Mo–.^22^ This situation allowed a tight connection between FeS_2_ and MoS_2_ and they were thus better able to interact with each other. After high temperature treatment, this special structure was partially broken to form FeS_2_ and MoS_2_. When the Fe concentration was too low, more MoS_2_ was formed and, meanwhile, some MoO_3_ and S vacancies also produced. When the Fe concentration was too high, more FeS_2_ was generated, but it easily combined with O_2_ in the air to form iron oxides like α-Fe_2_O_3_, due to the lack of Mo protection. The treatment temperature was another important factor for generating FeS_2_. When the temperature was too low, the above special structure was not decomposed to FeS_2_. When the temperature was too high, the catalyst was oxidized such that its structure was seriously destroyed. In addition, higher temperature could generate more S vacancies and the existence of S vacancies induced higher Fe^2+^/Fe^3+^ and Mo^4+^/Mo^6+^.

**Fig. 9:**
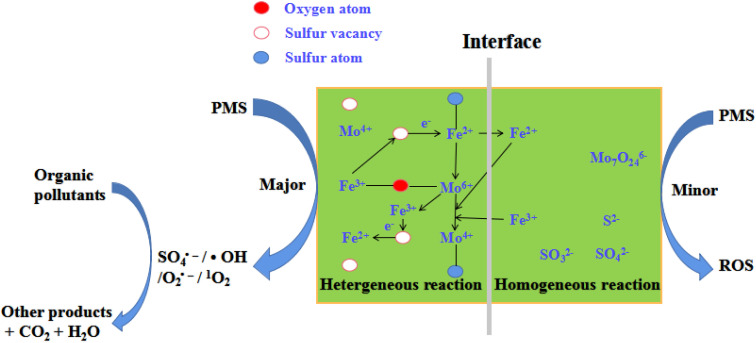
Schematic illustration of the removal of organic pollutants in the FeMoS_2_-IS/PMS system.

Based on the above results, a feasible reaction mechanism was proposed (Fig. 9). This was a typical heterogeneous catalytic reaction, which was different from that of the literature.^20–23,46–51^ The solution exhibited acidity during reaction and thus solution H^+^ corroded FeS_2_ and MoS_2_ on the surface of FeMoS_2_-IS to expose more Fe^2+^ and Mo^4+^ to generate SO_4_˙^−^ and ˙OH by the activation of PMS.^13–23^. Simultaneously, the catalyst could adsorb these ions by electrostatic interactions. Thus, these were coordination with Mo species to form Fe^2+^/Fe^3+^ and Mo^4+^/Mo^6+^ cycles, thereby accelerating PMS decomposition toward O_2_˙^−^ and ^1^O_2_. In addition, FeMoS_2_-IS was rich in S vacancies and these sites had good affinity for PMS, which then reacted with each other to form intermediates.^35^ At the liquid–solid interface, these intermediates finally evolved into O_2_˙^−^ and ^1^O_2_. Also, O_2_˙^−^, as an intermediate, reacted with each other to generate ^1^O_2_.^20^ In addition, the surface Mo^6+^ on the catalyst could react with O_2_˙^−^ to produce ^1^O_2_.^20^ Notably, the Mo^6+^ peroxo complex species could generate ^1^O_2_.^14^ The above pathways were displayed in formulas (6)–(17).

6 Fe^3+^ + Mo^4+^ → Fe^2+^ + Mo^6+^

7 Fe^2+^ + O_2_ + e^−^ → Fe^3+^ + O_2_˙^−^

8 Fe^3+^/Mo^6+^ + HSO_5_^−^ → Fe^2+^/Mo^4+^ + SO_5_˙^−^ + H^+^

9 2SO_5_˙^−^ + H_2_O → 2SO_4_^−^ + ^1^O_2_ + H^+^

10 FeMoS_2_-IS (vacancies) + PMS + e^−^ → PMS*–FeMoS_2_ (vacancies)

11 PMS*–FeMoS_2_ (vacancies) + H_2_O_buck_ → FeMoS_2_ (vacancies)–H_2_O_interface_ + O_2_˙^−^/^1^O_2_

12 2O_2_˙^−^ + H_2_O → H_2_O_2_ + OH^−^ + ^1^O_2_

13 Mo^6+^ + O_2_˙^−^ → Mo^4+^ + ^1^O_2_

14 MoO_3_ + H^+^ → HMoO_3_^+^

15 HMoO_3_^+^ + 2H_2_O_2_ → MoO(O_2_)_2_ + H^+^ + H_2_O

16 MoO(O_2_)_2_ + H_2_O → MoO(OH)(O_2_)_2_^−^ + H^+^

17 MoO(OH)(O_2_)_2_^−^ + H_2_O → MoO_4_^2−^ + ^1^O_2_ + H^+^

On all accounts, Fe, Mo, and S played important roles in the structure and catalytic performance of catalyst. First, for Fe ions, Fe^2+^ acted as an important active site for the formation of FeS_2_ and the activation PMS/dissolved O_2_ to generate ROS. Second, the effect of Mo mainly involved in this reaction and promoted RhB removal. Finally, the role of S suppressed the oxidation of low-valent Fe and Mo to high-valent metal ions. In addition, it provided a source of S deficient sites and could directly adsorb and activate PMS.

## 4. Conclusions

In summary, new Fe–Mo microparticles containing S vacancies with FeS_2_ as the dominant phases were successfully synthesized by the *in situ* synthesis. The catalyst effectively removed RhB by PMS activation in wastewater. High-temperature treatment and doping Fe were both beneficial for the formation of active sites, such as FeS_2_ and S vacancies. This system could effectively eliminate RhB over a wide pH range. All cations had positive effects on the RhB degradation but anions exhibited a dual effect. After operating through several reaction and regeneration cycles, the removal of RhB was still good. It was confirmed that SO_4_˙^−^, ˙OH, O_2_˙^−^ and ^1^O_2_ were important ROS in this reaction system, providing a good pathway for degrading organic pollutants in wastewater.

## Conflicts of interest

There are no conflicts to declare.

## Supplementary Material

RA-013-D3RA00707C-s001

## References

[cit1] Li S., Wu Y. N., Zheng H. S., Li H. B., Zheng Y. J., Nan J., Ma J., Nagarajand D., Chang J. S. (2023). Antibiotics degradation by advanced oxidation process (AOPs): recent advances in ecotoxicity and antibiotic-resistance genes induction of degradation products. Chemosphere.

[cit2] Thiam A., Sirés I., Brillas E. (2015). Treatment of a mixture of food color additives (E122, E124 and E129) in different water matrices by UVA and solar photoelectro-Fenton. Water Res..

[cit3] Yang Y. T., Chen J., Chen Z., Yu Z., Xue J. C., Luan T. G., Chen S. S., Zhou S. G. (2022). Mechanisms of polystyrene microplastic degradation by the microbially driven Fenton reaction. Water Res..

[cit4] Yang Z., Shan C., Pan B., Pignatello J. J. (2021). The Fenton reaction in water assisted by picolinic acid: accelerated iron cycling and co-generation of a selective Fe-based oxidant. Environ. Sci. Technol..

[cit5] Zhang T., Chen Y., Leiknes T. O. (2016). Oxidation of refractory benzothiazoles with PMS/CuFe_2_O_4_: kinetics and transformation intermediates. Environ. Sci. Technol..

[cit6] Li H., Shan C., Pan B. (2018). Fe(iii)-doped g-C_3_N_4_ mediated peroxymonosulfate activation for selective degradation of phenolic compounds *via* high-valent iron-oxo species. Environ. Sci. Technol..

[cit7] Gao Y., Zhu Y., Tong L., Chen Z., Jiang Q., Zhao Z., Liang X., Hu C. (2021). Unraveling the high-activity origin of single-atom iron catalysts for organic pollutant oxidation *via* peroxymonosulfate activation. Environ. Sci. Technol..

[cit8] So H.-L., Lin K.-Y., Chu W., Gong H. (2020). Degradation of triclosan by recyclable MnFe_2_O_4_-activated PMS: process modification for reduced toxicity and enhanced performance. Ind. Eng. Chem. Res..

[cit9] Zuo S., Li D., Guan Z., Fan Y., Xu H., Xia D., Wan J. (2021). Tailored d-band facilitating in Fe gradient doping CuO boosts peroxymonosulfate activation for high efficiency generation and release of singlet oxygen. ACS Appl. Mater. Interfaces.

[cit10] Wu L., Sun Z., Zhen Y., Zhu S., Yang C., Lu J., Tian Y., Zhong D., Ma J. (2021). Oxygen vacancy-Induced nonradical degradation of organics: critical trigger of oxygen (O_2_) in the Fe–Co LDH/peroxymonosulfate system. Environ. Sci. Technol..

[cit11] Li C., Yang S., Bian R., Tan Y., Dong X., Zhu N., He X., Zheng S., Sun Z. (2021). Clinoptilolite mediated activation of peroxymonosulfate through spherical dispersion and oriented array of NiFe_2_O_4_: upgrading synergy and performance. J. Hazard. Mater..

[cit12] Zhao Y., An H., Jing F., Ren Y., Ma J. (2019). Impact of crystal types of AgFeO_2_ nanoparticles on the peroxymonosulfate activation in the water. Environ. Sci. Technol..

[cit13] Yi Q., Liu W., Tan J., Yang B., Xing M., Zhang J. (2020). Mo^0^ and Mo^4+^ bimetallic reactive sites accelerating Fe^2+^/Fe^3+^ cycling for the activation of peroxymonosulfate with significantly improved remediation of aromatic pollutants. Chemosphere.

[cit14] Zhang Y., Niu J., Xu J. (2020). Fe(ii)-promoted activation of peroxymonosulfate by molybdenum disulfide for effective degradation of acetaminophen. Chem. Eng. J..

[cit15] Sheng B., Yang F., Wang Y., Wang Z., Qian L., Guo Y., Lou X., Liu J. (2019). Pivotal roles of MoS_2_ in boosting catalytic degradation of aqueous organic pollutants by Fe(ii)/PMS. Chem. Eng. J..

[cit16] Wang S., Xu W., Wu J., Gong Q., Xie P. (2020). Improved sulfamethoxazole degradation by the addition of MoS_2_ into the Fe^2+^/peroxymonosulfate process. Sep. Purif. Technol..

[cit17] He D., Cheng Y., Zeng Y., Luo H., Luo K., Li J., Pan X., Barceló D., Crittenden J. C. (2020). Synergistic activation of peroxymonosulfate and persulfate by ferrous ion and molybdenum disulfide for pollutant degradation: theoretical and experimental studies. Chemosphere.

[cit18] Yu L., Feng Y., Yang B., Yang Z., Shih K. (2021). Activation of peroxymonosulfate by molybdenum disulfide-mediated traces of Fe(iii) for sulfadiazine degradation. Chemosphere.

[cit19] Li X., Guo Y., Yan L., Yan T., Wen S., Feng R., Zhao Y. (2022). Enhanced activation of peroxymonosulfate by ball-milled MoS_2_ for degradation of tetracycline: boosting molybdenum activity by sulfur vacancies. Chem. Eng. J..

[cit20] Lu J., Zhou Y., Zhou Y. (2021). Efficiently activate peroxymonosulfate by Fe_3_O_4_@MoS_2_ for rapid degradation of sulfonamides. Chem. Eng. J..

[cit21] Yi C., He Z., Hu Y., Liang D., Zhang Y., Chen Y. (2021). FeOOH@MoS_2_ as a highly effective and stable activator of peroxymonosulfate-based advanced oxidation processes for pollutant degradation. Surf. Int..

[cit22] Zhou J., Guo X., Zhou X., Yang J., Yu S., Niu X., Chen Q., Li F., Liu Y. (2022). Boosting the efficiency of Fe-MoS_2_/peroxymonosulfate catalytic systems for organic pollutants remediation: insights into edge-site atomic coordination. Chem. Eng. J..

[cit23] Sun Y., Li R., Song C., Zhang H., Cheng Y., Nie A., Li H., Dionysiou D. D., Qian J., Pan B. (2021). Origin of the improved reactivity of MoS_2_ single crystal by confining lattice Fe atom in peroxymonosulfate-based Fenton-like reaction. Appl. Catal., B.

[cit24] Karroua M., Ladrière J., Matralis H., Grange P., Delmon B. (1992). Characterisation of unsupported FeMoS catalysts: stability during reaction and effect of the sulfiding temperature. J. Catal..

[cit25] Zhao X., Xiao M., Lu Q., Li Q., Han C., Xing Z., Yang X. (2017). FeS_2_-doped MoS_2_ nanoflower with the dominant 1T-MoS_2_ phase as an excellent electrocatalyst for high-performance hydrogen evolution. Electrochim. Acta.

[cit26] Zhou Y., Wang X. L., Zhu C. Y., Dionysiou D. D., Zhao G. C., Fang G. D., Zhou D. M. (2018). New insight into the mechanism of peroxymonosulfate activation by sulfur containing minerals: role of sulfur conversion in sulfate radical generation. Water Res..

[cit27] Liu X. W., Li H., Gao S., Bai Z., Tian J. (2022). Peroxymonosulfate activation by different iron sulfides for bisphenol-A degradation: performance and mechanism. Sep. Purif. Technol..

[cit28] Huang M., Wang X., Liu C., Fang G., Gao J., Wang Y., Zhou D. (2021). Mechanism of metal sulfides accelerating Fe(ii)/Fe(iii) redox cycling to enhance pollutant degradation by persulfate: metallic active sites *vs.* reducing sulfur species. J. Hazard. Mater..

[cit29] Wu L., Guo P., Wang X., Li H., Li A., Chen K. (2022). Mechanism study of CoS_2_/Fe(iii)/peroxymonosulfate catalysis system: the vital role of sulfur vacancies. Chemosphere.

[cit30] Liang C., Huang C.-F., Mohanty N., Kurakalva R. M. (2008). A rapid spectrophotometric determination of persulfate anion in ISCO. Chemosphere.

[cit31] Chang L., Yi H., Yang B., Jia F., Song S. (2021). Activation of Fenton reaction by controllable oxygen incorporation in MoS_2_-Fe under visible light irradiation. Appl. Surf. Sci..

[cit32] Aseman-Bashiz L. E., Sayyaf H. (2021). Synthesis of nano-FeS_2_ and its application as an effective activator of ozone and peroxydisulfate in the electrochemical process for ofloxacin degradation: a comparative study. Chemosphere.

[cit33] Ma X., Cheng Y., Deng J., Cai A., Xiao L., Li J., Li X. (2021). Elucidating the role of Fe(iv) and radical species for CBZ degradation in FeS_2_/PS system. Sep. Purif. Technol..

[cit34] Zhou H., Lai L., Wan Y., He Y., Yao G., Lai B. (2022). Molybdenum disulfide (MoS_2_): a versatile activator of both peroxymonosulfate and persulfate for the degradation of carbamazepine. Chem. Eng. J..

[cit35] Yang J.-C. E., Zhu M.-P., Duan X., Wang S., Yuan B., Fu M.-L. (2021). The mechanistic difference of 1T–2H MoS_2_ homojunctions in persulfates activation: structure dependent oxidation pathways. Appl. Catal., B.

[cit36] Du M., Yi Q., Ji J., Zhu Q., Duan H., Xing M., Zhang J. (2020). Sustainable activation of peroxymonosulfate by the Mo(iv) in MoS_2_ for the remediation of aromatic organic pollutants. Chin. Chem. Lett..

[cit37] da Silveira Salla J., da Boit Martinello K., Dotto G. L., García-Díaz E., Javed H., Alvarez P. J. J., Luiz Foletto E. (2020). Synthesis of citrate-modified CuFeS_2_ catalyst with significant effect on the photo-Fenton degradation efficiency of bisphenol A under visible light and near neutral pH. Colloids Surf., A.

[cit38] Tian S. H., Tu Y. T., Chen D. S., Chen X., Xiong Y. (2011). Degradation of acid orange II at neutral pH using Fe_2_(MoO_4_)_3_ as a heterogeneous Fenton-like catalyst. Chem. Eng. J..

[cit39] Huang Y. C., Lai L., Huang W., Zhou H., Li J., Liu C., Lai B., Li N. (2022). Effective peroxymonosulfate activation by natural molybdenite for enhanced atrazine degradation: role of sulfur vacancy, degradation pathways and mechanism. J. Hazard. Mater..

[cit40] Kuang H., He Z., Mu L., Huang R., Zhang Y., Xu X., Wang L., Chen Y., Zhao S. (2021). Enhancing co-catalysis of MoS_2_ for persulfate activation in Fe^3+^-based advanced oxidation processes *via* defect engineering. Chem. Eng. J..

[cit41] Luo H., Cheng Y., Zeng Y., Luo K., He D., Pan X. (2020). Rapid removal of organic micropollutants by heterogeneous peroxymonosulfate catalysis over a wide pH range: performance, mechanism and economic analysis. Sep. Purif. Technol..

[cit42] Mehdi A., Ghanbari F. (2019). Organic dye degradation through peroxymonosulfate catalyzed by reusable graphite felt/ferriferrous oxide: mechanism and identification of intermediates. Mater. Res. Bull..

[cit43] Nematollah J., Ghanbari F., Amir Z. (2018). Coupling electro-oxidation and oxone for degradation of 2,4-dichlorophenoxyacetic acid (2,4-D) from aqueous solutions. J. Water Proc. Eng..

[cit44] Song X., Ni J., Liu D., Shi W., Yuan Y., Cui F., Tian J., Wang W. (2022). Molybdenum disulfide as excellent co-catalyst boosting catalytic degradation of sulfamethoxazole by *n*ZVI/PDS process. Sep. Purif. Technol..

[cit45] Rashad M. M., Ibrahim A. A., Rayan D. A., Sanad M. M. S., Helmy I. M. (2017). Photo-Fenton-like degradation of rhodamine B dye from waste water using iron molybdate catalyst under visible light irradiation. Environ. Nanotechnol., Monit. Manage..

[cit46] Oh S.-Y., Kang S.-G., Kim D.-W., Chiu P. C. (2011). Degradation of 2,4-dinitrotoluene by persulfate activated with iron sulfides. Chem. Eng. J..

[cit47] Zheng X., Niu X., Zhang D., Ye X., Ma J., Lv M., Zhang L. (2022). Removal of *Microcystis aeruginosa* by natural pyrite-activated persulfate: performance and the significance of iron species. Chem. Eng. J..

[cit48] Peng H., Zhu J., Chen Y., Chen F., Zhu J., Liu M., Zhang K., Gan M. (2021). Pyrite-activated persulfate for simultaneous 2,4-DCP oxidation and Cr(vi) reduction. Chem. Eng. J..

[cit49] Diao Z.-H., Liu J.-J., Hu Y.-X., Kong L.-J., Jiang D., Xu X.-R. (2017). Comparative study of rhodamine B degradation by the systems pyrite/H_2_O_2_ and pyrite/persulfate: reactivity, stability, products and mechanism. Sep. Purif. Technol..

[cit50] Sühnholz S., Kopinke F.-D., Mackenzie K. (2020). Reagent or catalyst? FeS as activator for persulfate in water. Chem. Eng. J..

[cit51] Wang X. B., Wang Y., Chen N., Shi Y., Zhang L. (2020). Pyrite enables persulfate activation for efficient atrazine degradation. Chemosphere.

[cit52] Zhou L., Song W., Chen Z. Q., Yin G. C. (2013). Degradation of organic pollutants in wastewater by bicarbonate activated hydrogen peroxide with a supported cobalt catalyst. Environ. Sci. Technol..

[cit53] Heckert E. G., Seal S., Self W. T. (2008). Fenton-like reaction catalyzed by the rare earth inner transition metal cerium. Environ. Sci. Technol..

