# Supplementary material for: FeMoS2 micoroparticles as an excellent catalyst for the activation of peroxymonosulfate toward organic contaminant degradation
Source: RSC Adv. 2023 Mar 17;13(13):8720–35. doi: 10.1039/d3ra00707c (PMC10020838; doi:10.1039/d3ra00707c)
Supplement: RA-013-D3RA00707C-s001 [file RA-013-D3RA00707C-s001.pdf]

Electronic Supplementary Material (ESI) for RSC Advances.
This journal is © The Royal Society of Chemistry 2023

Support Materials

$FeMoS_2$ Micoroparticles as an Excellent Catalyst for the Activation of Peroxymonosulfate toward Organic Contaminant Degradation

Cai-Wu Luo, [a, b, *] Lei Cai, [b] Chao Xie, [b] Jing Wu, [c] Tian-Jiao Jiang [b]

[a] Research Center for Eco-Environmental Sciences, Chinese Academy of Sciences, 100085, China

[b] School of Resource Environmental and Safety Engineering, University of South China, 421000, China

[c] Ningxia Modern Construction Technology Vocational Skills Public Training Center, Ningxia College of Construction, 750021, China

* Corresponding author: Cai-Wu Luo;

Tel: +86-734-8282345;

E-mail address: luocaiwu00@126.com.

Figure S1

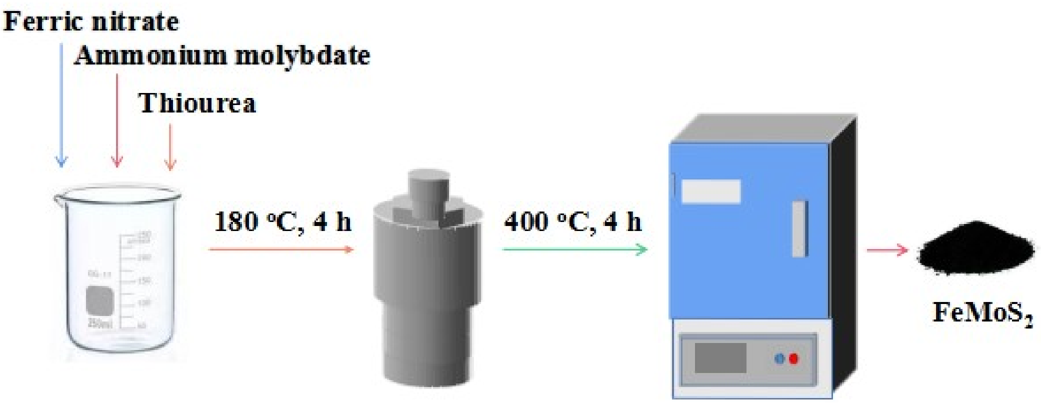

Figure S1 Synthetic route of $FeMoS_2$ microparticles.

Figure S2

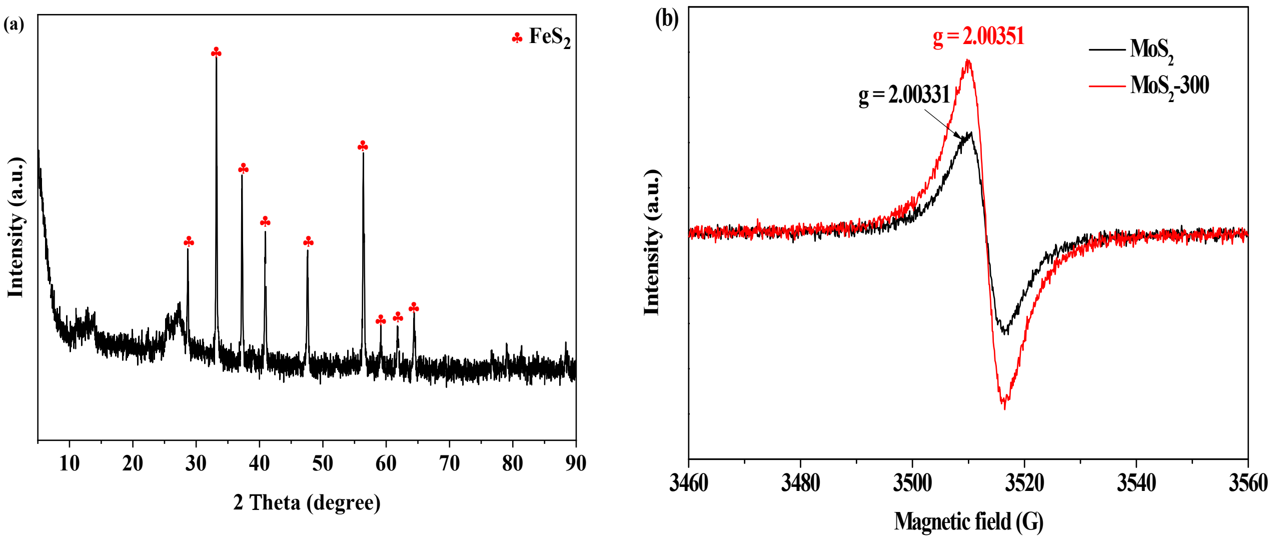

Figure S2 (a) XRD pattern of Fe-S catalyst; (b) EPR signal of sulfur vacancies in MoS_2 and MoS_2-300.

Figure S3

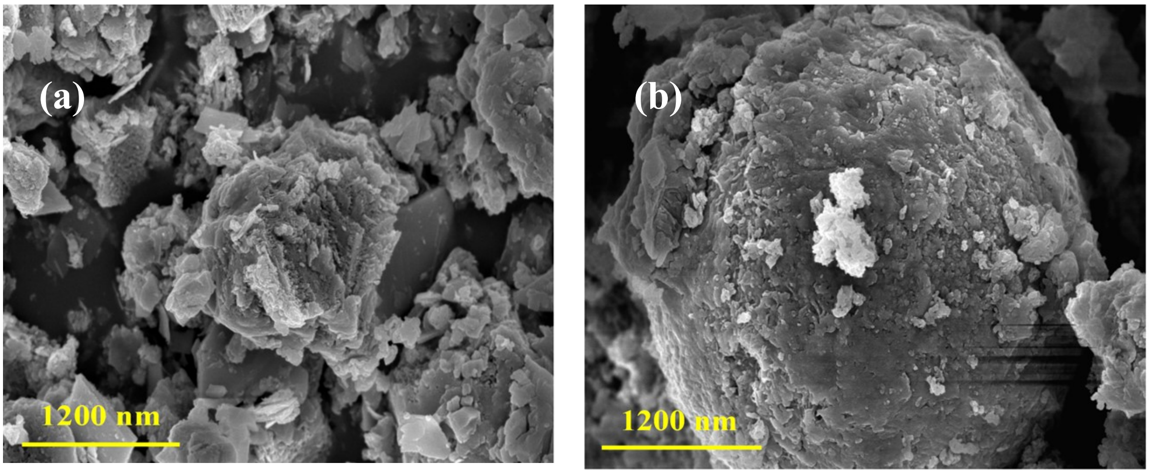

Figure S3 (a) SEM image of MoS_2-IS; (b) SEM image of $FeMoS_2$-IS.

Figure S4

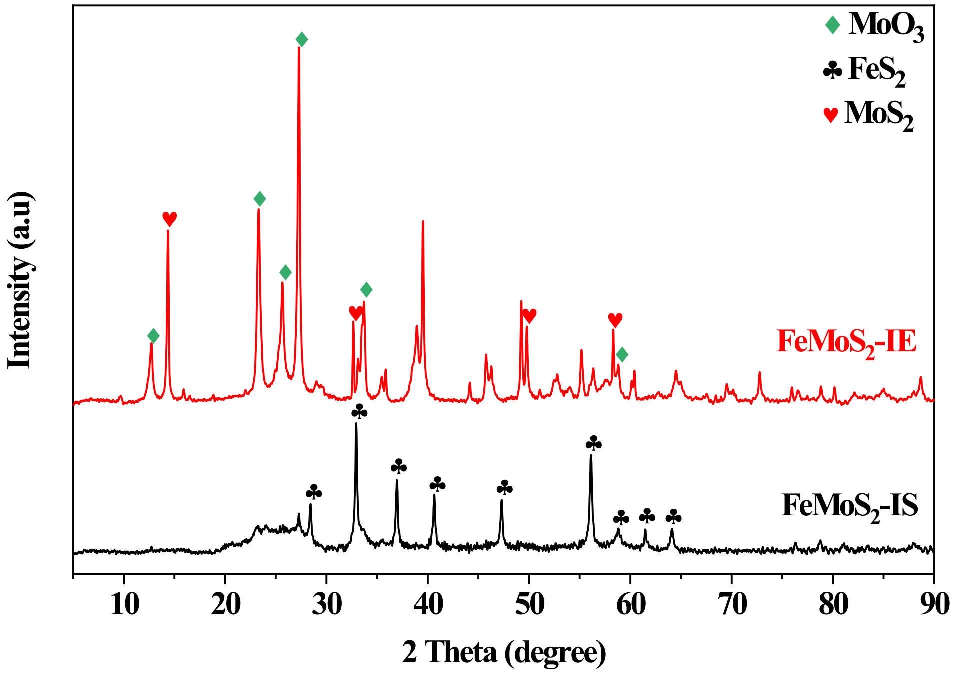

Figure S4 XRD patterns of $FeMoS_2$-IS and $FeMoS_2$-IE.

Figure S5

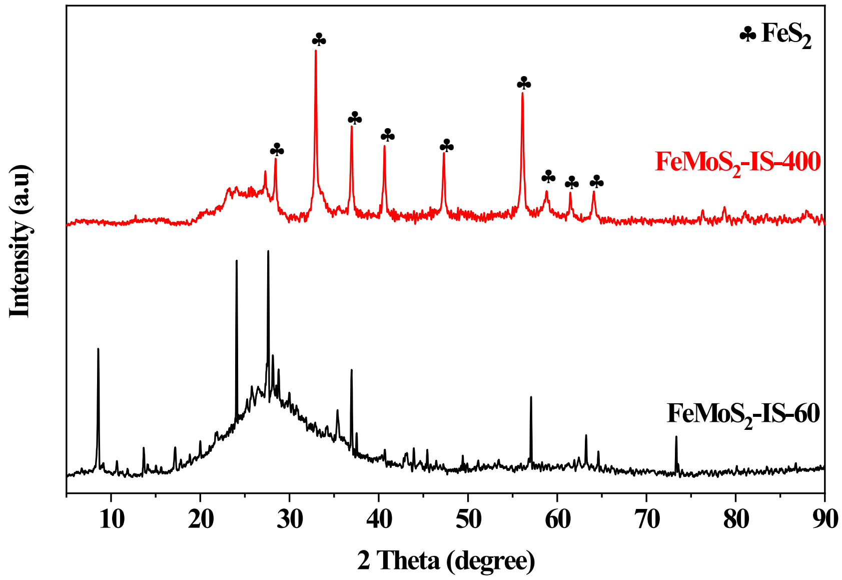

Figure S5 XRD patterns of $FeMoS_2$-IS-60 and $FeMoS_2$-IS-400.

Figure S6

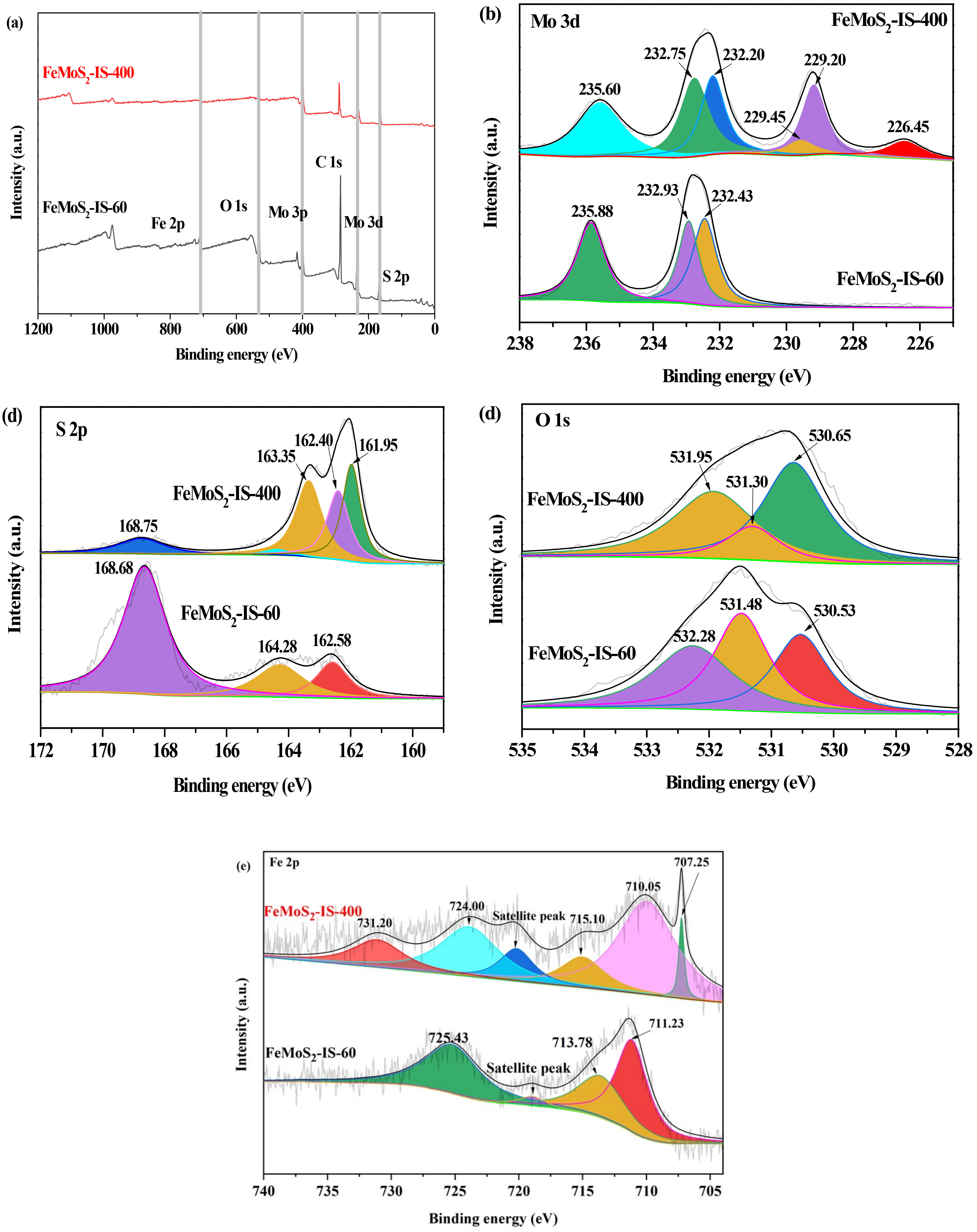

Figure S6 XPS spectra of (a) survey spectra, (b) Mo 3d, (c) S 2p, (d) O 1s and (e) Fe 2p in the $FeMoS_2$-IS-60 and $FeMoS_2$-IS-400.

Figure S7

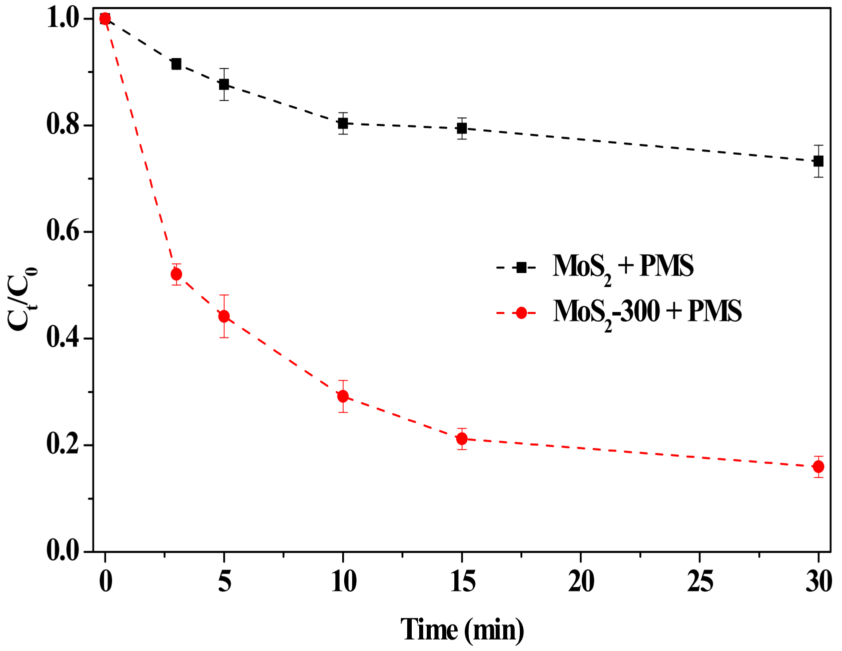

Figure S7 Removal of RhB over MoS_2 and MoS_2-300 activating PMS. Reaction conditions: [RhB] = 10 mg/L, initial pH = 3.0, [MoS_2 and MoS_2-300] = 1.0 g/L, [PMS] = 1.0 mM and in the darkness.

Figure S8

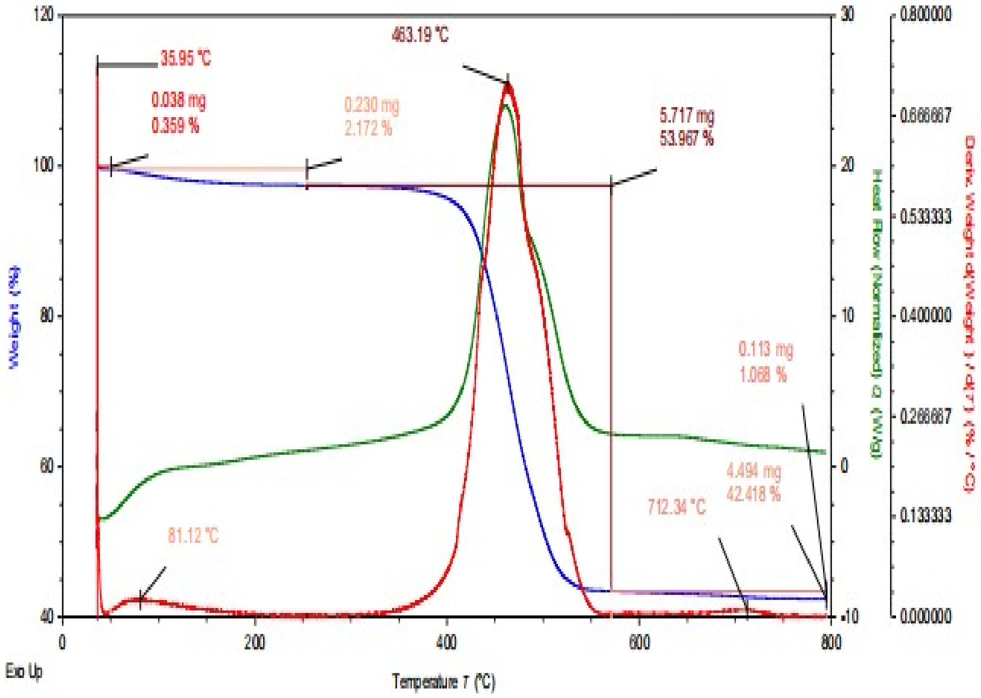

Figure S8 TG-DSC of $FeMoS_2$-IS.

Figure S9

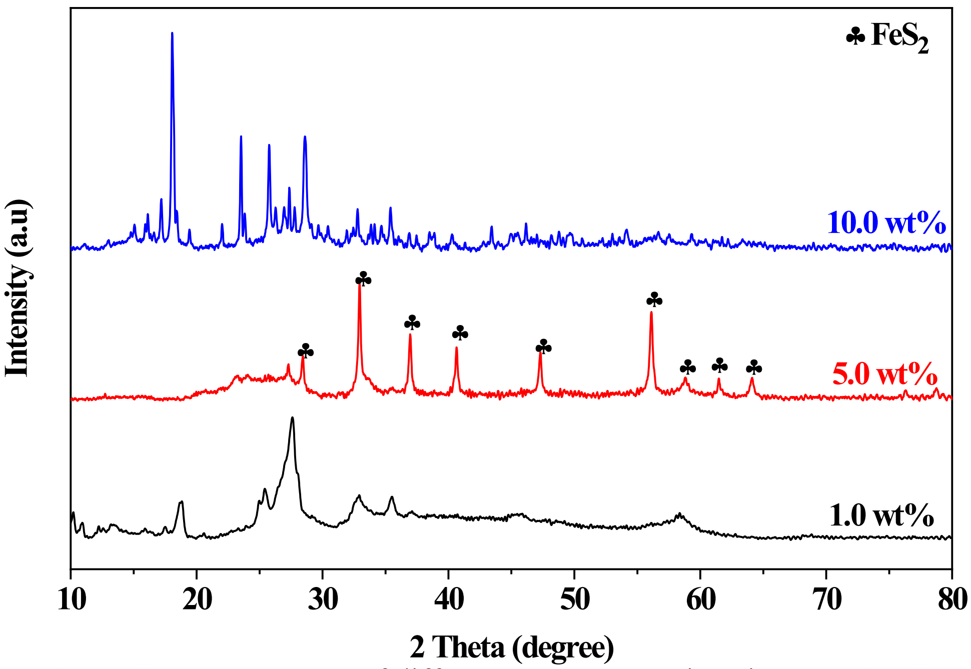

Figure S9 XRD patterns of different Fe concentrations in $FeMoS_2$-IS.

Figure S10

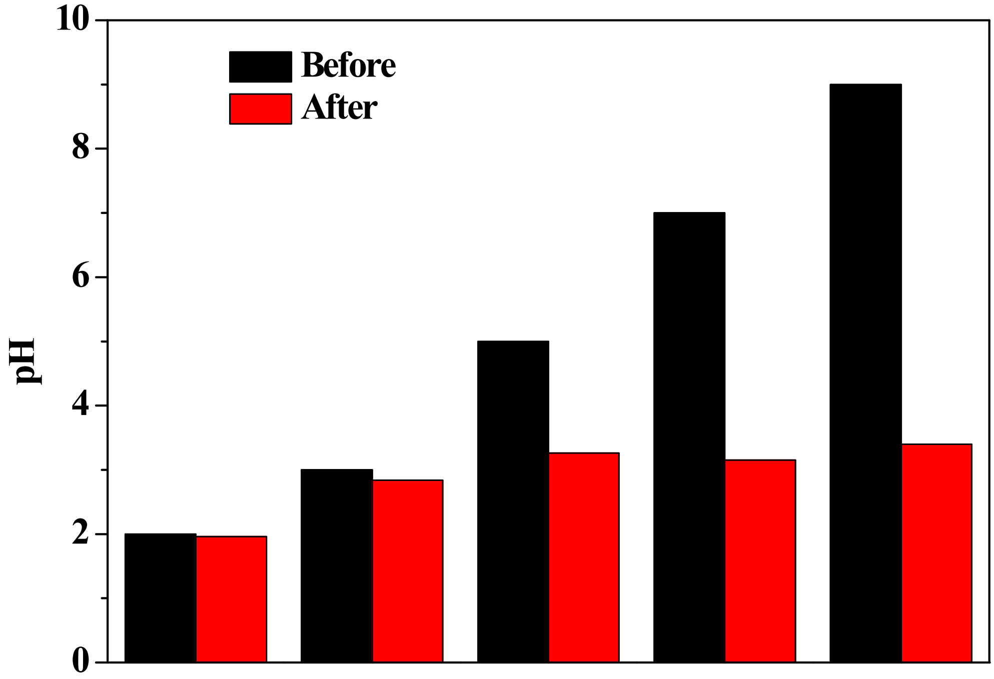

Figure S10 Change of solution pH before and after the reaction. Reaction conditions: [RhB] = 10 mg/L, [$FeMoS_2$-IS] = 1.0 g/L, [PMS] = 1.0 mM and in the darkness.

Figure S11

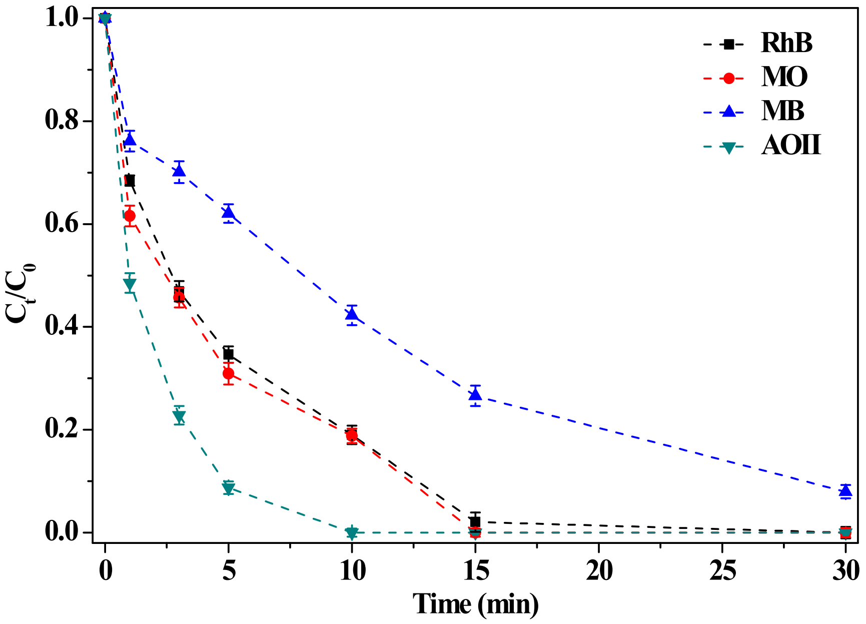

Figure S11 Removal of different organic pollutants. Reaction conditions: [Organic pollutants] = 20.8 μM, initial pH = 3.0, [$FeMoS_2$-IS] = 0.3 g/L, [PMS] = 1.0 mM and in the darkness.

Figure S12

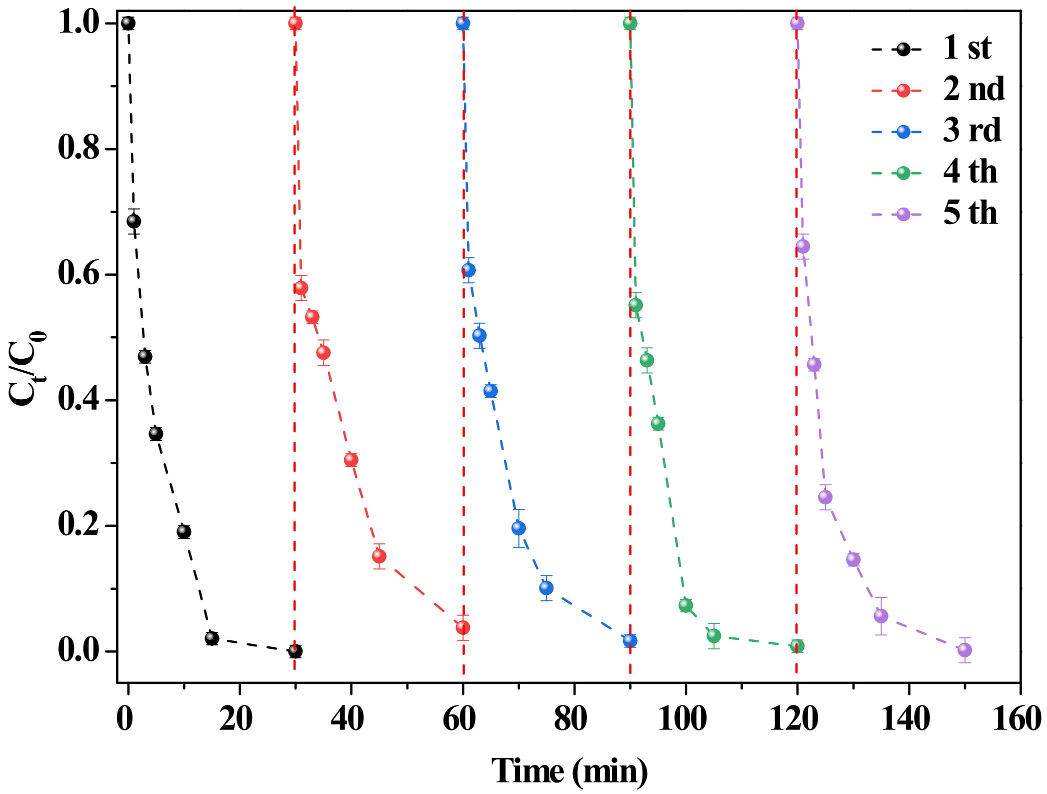

Figure S12 The stability of catalyst. Reaction conditions: [RhB] = 10 mg/L, initial pH = 3.0, [$FeMoS_2$-IS] = 0.3 g/L, [PMS] = 1.0 mM and in the darkness.

Figure S13

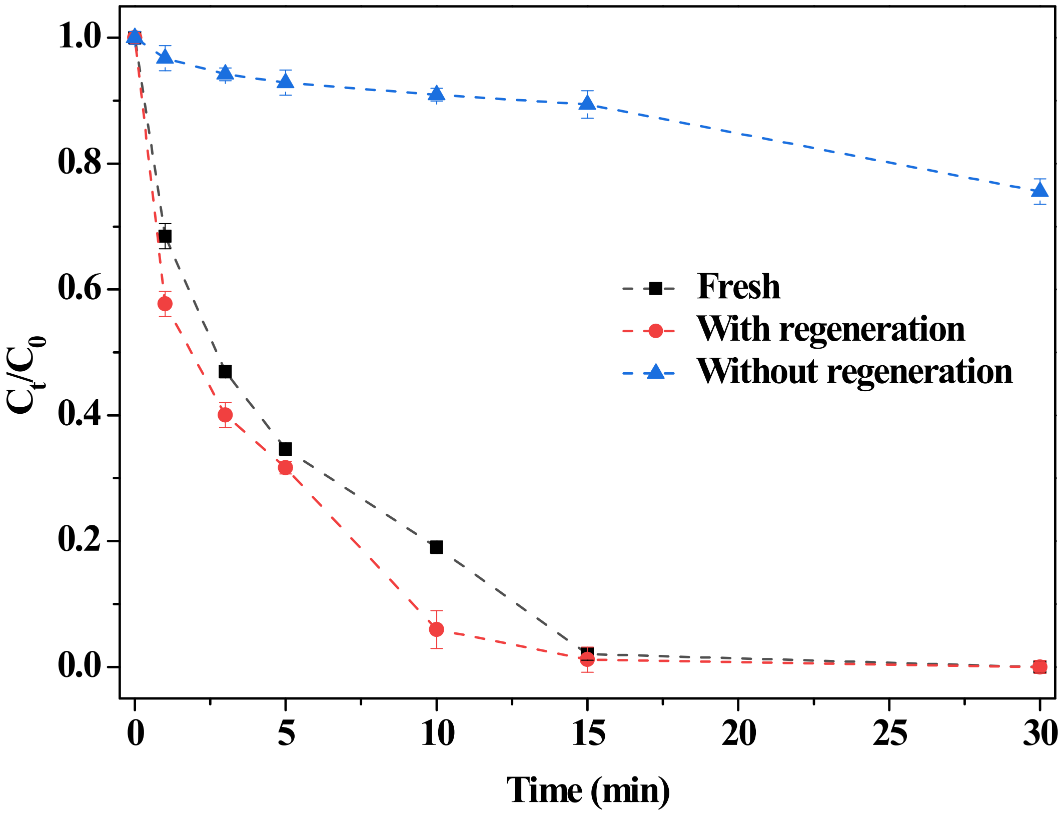

Figure S13 Comparison of regeneration and without regeneration on the removal of RhB. Reaction conditions: [RhB] = 10 mg/L, initial pH = 3.0, [$FeMoS_2$-IS] = 0.3 g/L, [PMS] = 1.0 mM and in the darkness.

Figure S14

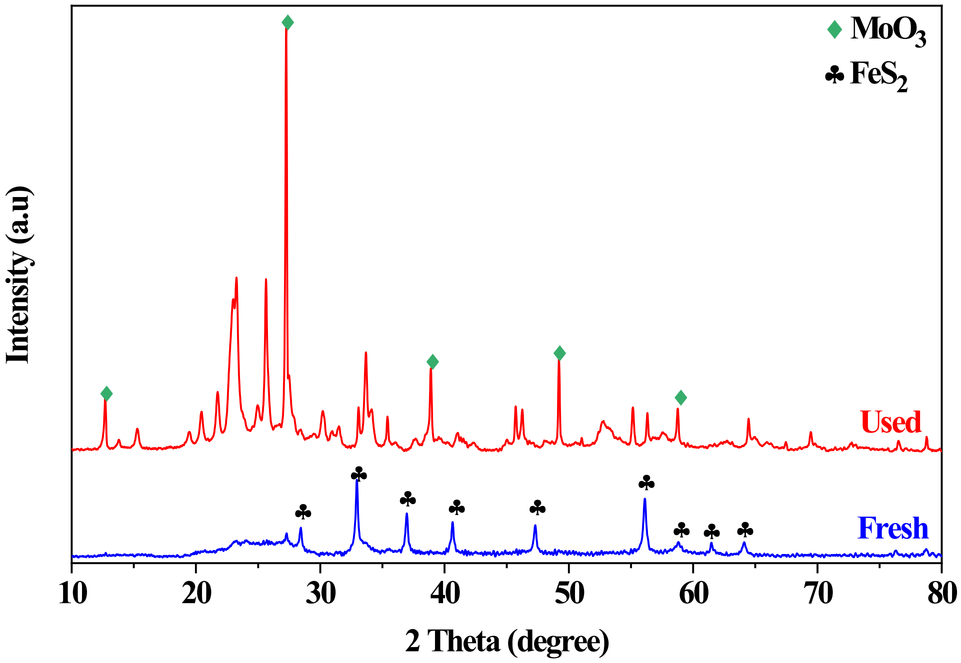

Figure S14 XRD patterns of fresh and used $FeMoS_2$-IS.

Figure S15

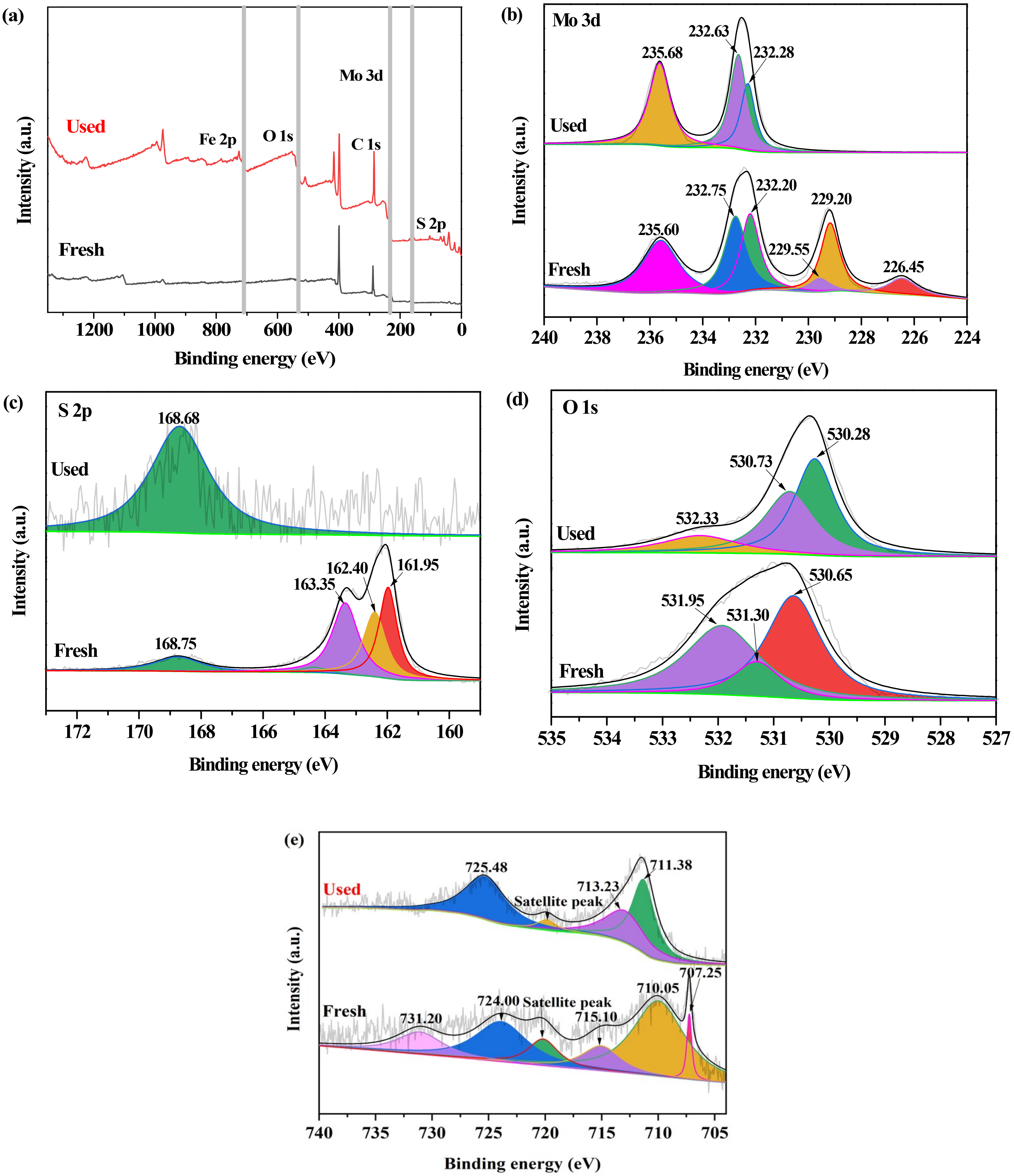

Figure S15 XPS spectra of (a) survey spectra, (b) Mo 3d, (c) S 2p, (d) O 1s and (e) Fe 2p in the fresh and used $FeMoS_2$-IS.

Figure S16

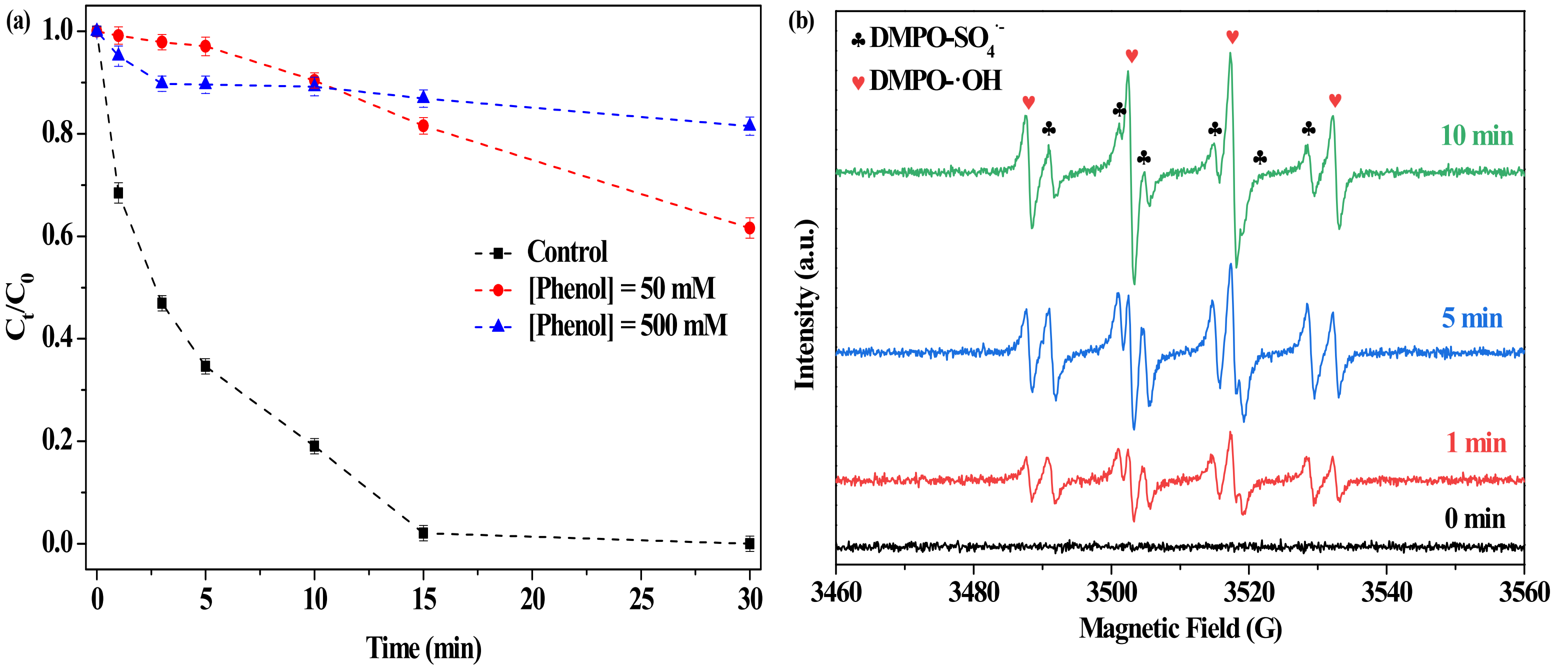

Figure S16 Effect of phenol (a) on the removal of RhB in the $FeMoS_2$-IS/PMS process and signals of EPR (b). Reaction conditions: [RhB] = 10 mg/L, initial pH = 3.0, [$FeMoS_2$-IS] = 0.3 g/L, [PMS] = 1.0 mM and in the darkness.

Table S1 Changes of surface elements from XPS characterization.

Catalysts	Mo^{4+}/Mo^{6+}	2H-/1T-MoS_2	Mo^{6+} [a]	Mo-S	Fe^{2+} [b]	Fe^{2+}/Fe^{3+}
MoS_2-IS	2.23	1.18	0.31	0.031	No [c]	No
$FeMoS_2$-IS	2.56	0.74	0.28	0.068	0.67	1.99
$FeMoS_2$-IS-60	0.56	0.81	0.36	No	0.40	0.65
$FeMoS_2$-IS [d]	1.46	1.63	0.41	No	0.38	0.62

[a]: $Mo^{6+}/(Mo^{4+} + Mo^{6+})$; [b]: $Fe^{2+}/(Fe^{2+} + Fe^{3+})$; [c] No wasn't detected; [d]: after five runs. All values were based on the peak of each element from XPS characterization.
